# Curcumin and its Derivatives: Their Application in Neuropharmacology and Neuroscience in the 21st Century

**DOI:** 10.2174/1570159X11311040002

**Published:** 2013-07

**Authors:** Wing-Hin Lee, Ching-Yee Loo, Mary Bebawy, Frederick Luk, Rebecca S Mason, Ramin Rohanizadeh

**Affiliations:** 1 Advanced Drug Delivery Group, Faculty of Pharmacy, University of Sydney, NSW 2006, Australia;; 2 School of Pharmacy, Graduate School of Health, University of Technology Sydney PO Box 123 Broadway, NSW 2007, Australia;; 3 Physiology and Bosch Institute, University of Sydney, NSW 2006, Australia

**Keywords:** Curcumin, Alzheimer, Parkinson, glioblastoma, anti-oxidant, anti-inflammatory, reactive oxygen species

## Abstract

Curcumin (diferuloylmethane), a polyphenol extracted from the plant Curcuma longa, is widely used in Southeast Asia, China and India in food preparation and for medicinal purposes. Since the second half of the last century, this traditional medicine has attracted the attention of scientists from multiple disciplines to elucidate its pharmacological properties. Of significant interest is curcumin’s role to treat neurodegenerative diseases including Alzheimer’s disease (AD), and Parkinson’s disease (PD) and malignancy. These diseases all share an inflammatory basis, involving increased cellular reactive oxygen species (ROS) accumulation and oxidative damage to lipids, nucleic acids and proteins. The therapeutic benefits of curcumin for these neurodegenerative diseases appear multifactorial via regulation of transcription factors, cytokines and enzymes associated with (Nuclear factor kappa beta) NFκB activity. This review describes the historical use of curcumin in medicine, its chemistry, stability and biological activities, including curcumin's anti-cancer, anti-microbial, anti-oxidant, and anti-inflammatory properties. The review further discusses the pharmacology of curcumin and provides new perspectives on its therapeutic potential and limitations. Especially, the review focuses in detail on the effectiveness of curcumin and its mechanism of actions in treating neurodegenerative diseases such as Alzheimer’s and Parkinson’s diseases and brain malignancies.

## INTRODUCTION

Turmeric (*Curcuma longa*) is an intriguing ingredient with a rich history as a dietary spice and herbal supplement in ancient China and India [1]. This distinctive yellow- colored spice, derived from the rhizome of the plant (*C. longa*), is a member of Zingiberaceae family and is widely cultivated in India and Southeast Asia. The use of turmeric in Asia dates back more than 2000 years where it was used in cooking, medicine, cosmetics and fabric dyes. Tumeric only gained popularity in European society from the 14^th^ century A.D [1]. Turmeric is arguably one of the most widely used spices throughout the world, as evident from its numerous names as adopted by the locals; i.e. ukon, Indian saffron, kurkum, yellow ginger and kunyit basah. A rough estimate by Ravindran showed that at least 20 countries use turmeric in basic food preparation daily [2]. The primary active compound responsible for the yellow color named curcumin was identified in early 1900 by Lampe and Milobedzka [3]. The ‘magic touch’ of turmeric in curing various ailments due to its broad spectrum of pharmacological activities has been noted since ancient times. The ancient Indian holistic medicine known as Ayurveda uses turmeric for the treatment of common eye infections, burns, acne, wound dressing, sprains and swelling [4]. In traditional medicine turmeric is used to enhance the immune system and as a cure for different respiratory diseases such as asthma and for allergy [4]. Tumeric has also been traditionaly used for the treatment of diabetes, cough, sinusitis, flu, rheumatism and liver disorders [4]. Meanwhile, traditional Chinese medicine practitioners regularly use turmeric for treating abdominal pain associated diseases [5]. It has widely been accepted since ancient times that this polyphenol compound possesses anti-inflammatory properties [6]. Advancements in modern medicine have revealed many unkown medicinal properties of turmeric which include anti-oxidant, anti-mutagenic, anti-cancer, anti-microbial and anti-cardiovascular activities [7-3]. Studies have also strongly indicated that curcumin, the active compound in turmeric, is the key ingredient responsible for the major therapeutic activities of turmeric [14, 15]. Some differences in the applications of turmeric or curcumin in ancient period and in modern society are listed in Table **1**. 

Due to its multi-faceted pharmacology, concerted efforts have been made to evaluate the possibility of using curcumin for the treatment or prevention of neurodegenerative diseases such as Alzheimer’s disease (AD) [16, 17], Parkinson’s disease (PD) [18, 19] and brain tumors [20-23], which have so far demonstrated encouraging results. The brain is the most vulnerable organ to oxidative damage, as it is responsible for 20% of the body’s oxygen consumption despite accounting for only 2% of total body weight [24]. The presence of abundant oxidisable unsaturated fatty acids and redox-active transition metal ions also provides additional insult to oxidative damage in brain [24]. Oxidative stress and generated free radicals which mainly occur in the highly oxidative intracellular microenvironment surrounding the neurons and glial cells, are associated with brain ageing and brain injury [24]. Reactive oxygen species (ROS) also referred as pro-oxidant, are by-products of aerobic metabolism and are second messengers of transduction pathways [25]. Under normal physiological conditions, ROS are present in minimal concentrations and counter-balanced with appropriate amount of anti-oxidants. However, when the delicate balance between ROS and anti-oxidants is disrupted, the ensuing oxidative stress generates damaging effects to DNA, proteins and lipids that lead to progression of neurodegenerative diseases such as AD and PD. Recently, the term “nitrosative stress” or reactive nitrogen species (RNS) has been coined to indicate the damage caused by anion peroxynitrite, nitric oxide, nitroxyl anion and nitrosonium (extensively reviewed in [26]). In order for cells to survive the assault of ROS and RNS effectively, the generation of molecules equipped with anti-oxidant properties and perhaps anti-apoptotic activities are required. Enzymes such as heat shock protein-32, superoxide dismutase, glutathione peroxidases and catalase provide cells with the ability to combat the ROS and RNS. Besides that, exogenous anti-oxidants such as vitamins A, C and E [27, 28] and bio-macromolecules (flavonoids and curcumin) [29-31] and endogenous anti-oxidants (glutathione) are among candidates endowed with potent anti-oxidant properties. It is a known fact that curcumin is a strong anti-oxidant compound with great ability to scavenge the oxygen-derived free radicals. Consequently, curcumin could be a potential neuroprotective agent. 

The progression of AD is multifactorial and includes aggregation of amyloid-*β* peptide (A*β*), oxidative stress and reduction of acetylcholine (AChE) levels [32-34]. It is interesting to note that the outcome from an epidemiologic investigation in India showed that the occurrence of AD is the lowest in comparison with other countries, an observation which could be related to their high curcumin dietary consumption [35]. Cummulative scientific evidence from both *in vitro *and *in vivo *models has confirmed the role of curcumin in controlling AD by inhibiting the A*β*-plaque formation, through binding of curcumin to A*β* peptide and fibrils [36-41]. PD is the second most common neuro-degenerative disease in which the accumulation of aggregated α-synuclein is the main factor contributing to disease progression [42, 43]. The formation of nonfibrillar oligomer and protofibrillar structures of α-synuclein has been shown to cause apoptosis in cells [44]. The risk of PD is increased by exposure to neurotoxins and pesticides and through oxidative damage to neurons by a decrease in the mitochondrial complex I activity and glutathione levels [18, 45-49]. Curcumin decreases the cytotoxicity of aggregated α-synuclein towards neuroblastoma cells along with intracellular ROS levels [50]. In addition, curcumin is also effective in the treatment of brain malignancies such as glioma, pituitary adenoma and nerve sheath tumors [51-54]. Suppression of cancer activators such as NFκB serves to down regulate inflammatory cytokines, responsible for tumorigenesis [55].

This review describes the recent findings regarding the effectiveness of curcumin in combating neurodegenerative disorders including AD, PD and brain tumors. In addition, the key roles of curcumin in controlling the symptoms of these diseases are discussed in detail. Even though curcumins are versatile compounds with broad spectrum of therapeutic activities, the low bioavailability of curcumin together with its inherent insolubility remains a major hurdle which limits its mainstream application. 

## HISTORY OF CURCUMIN

### Chemical Properties of Curcumin

**Fig. (1):**
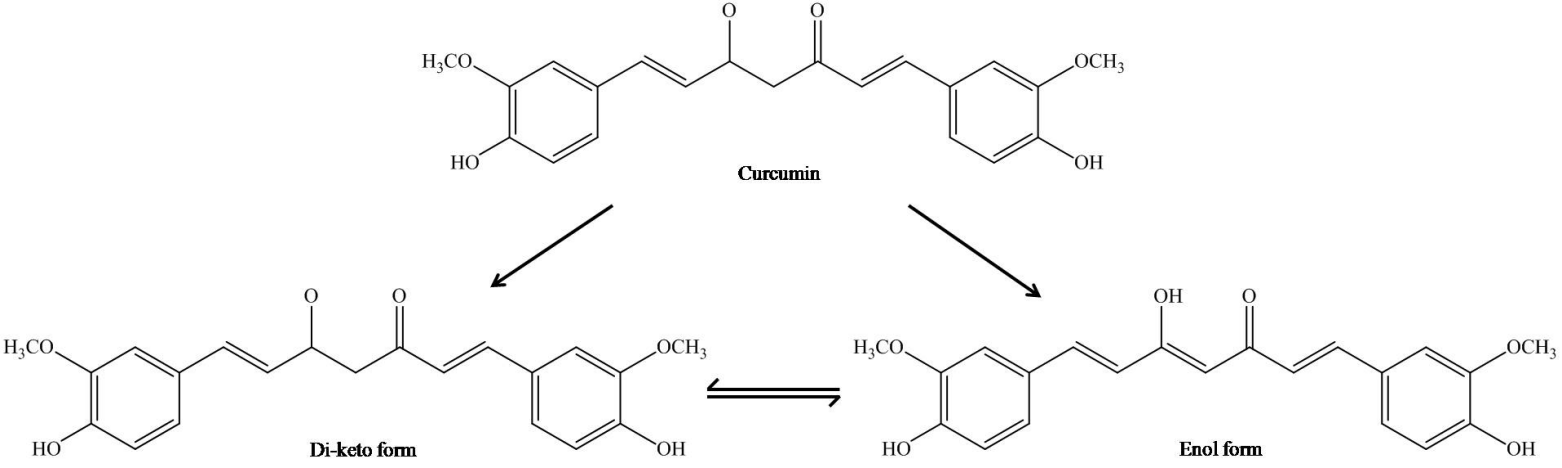
Chemical structure of curcumin existing in keto-enol tautomeric forms.

Curcumin, also known as diferuloylmethane (C_21_H_20_O_6_), is a low molecular mass (368.37 g/mol) polyphenol compound with a melting temperature of approximately 183 °C. The IUPAC name of curcumin is 1,7-bis(4-hydroxy-3-methoxy phenyl)-1,6-heptadiene-3,5-dione (1E-6E) where the two aryl rings containing ortho-methoxy phenolic OH^–^ groups are symmetrically linked to a β-diketone moiety (Fig. **1**). The occurrence of intramolecular hydrogen atoms transfer at the β-diketone chain of curcumin leads to the existence of keto and enol tautomeric conformations in equilibrium (Fig. **1**). In addition, these keto-enol tautomers also exist in several *cis* and *trans* forms whereby their relative concentrations vary according to temperature, polarity of solvent, pH and substitution of the aromatic rings [56-58]. The amount of keto-enol-enolate of the heptadienone moiety in equilibrium plays a crucial role in the physicochemical properties and anti-oxidant activities of curcumin [59]. In acidic and neutral conditions (i.e. pH 3–7), the major constituents present are curcumin molecules in bis-keto form where curcumin acts as a potent proton donor [60]. This is attributable to the presence of a highly activated carbon atom in the heptadienone linkage between the two-methoxy phenol rings of bis-keto form of curcumin. However, in situations (i.e. pH > 8) where the enolate form of the heptadienone chains predominates, curcumin acts instead as an electron donor. The presence of enolate in solution is found to be important in the radical-scavenging ability of curcumin. Curcumin has three ionisable protons contributed by the enolic proton (approximate pKa of 8.5) and two phenolic OH^–^ groups (pKa of 10–10.5) [61, 62].

Due to its hydrophobic nature, curcumin is poorly soluble in neutral solvent (i.e. water). Curcumin’s solubility is improved slightly in basic conditions [63]. However, curcumin is readily soluble in organic solvents such as ethanol, methanol, isopropanol, acetone and dimethylsulfoxide (DMSO) and has moderate solubility in hexane, cyclohexane, tetrahydrofuran and dioxane [63]. Curcumin exhibits strong absorption spectrophotometrically with the maximum absorption (λ_max_) ranging from 408–434 nm [64]. The absorption band of curcumin in most polar solvents such as methanol and DMSO is red-shifted and broad with λ_max_ at ~420 nm. In most hydrogen bond acceptor and proton donor solvents, λ_max_ is roughly centered at 430 to 434 nm, except for methanol where it absorbs maximally at 423–428 nm [65-67]. In non-polar solvents such as hexane and cyclohexane, the absorption spectra are blue-shifted. This could be attributable to the stabilizing effect of non-polar solvents towards the less polar bis-keto form of curcumin, which in turn elevates curcumin’s concentration in equilibrium. Hence, the blue-shifted and sharper peaks were observed in non-polar solvents. In contrast, the enolate form of curcumin predominates in polar solvents, resulting in the presence of broader absorption peaks (extensively reviewed in [63]). Comprehensive studies on the excitation state, fluorescence spectrum and maximal intensity of curcumin in different solvents, micro-heterogeneous systems and bio-mimetic systems showed high sensitivity and dependency towards the nature of solvents [63, 68, 69]. The fluorescence spectrum of curcumin in non-polar solvents (i.e. benzene) is sharp and peaked at ~460 nm. The fluorescence spectrum in cyclohexane is unique compared to other non-polar solvents where it has two maximal fluorescence peaks at 446 and 470 nm [66, 69]. Meanwhile, the maximal fluorescence intensities for aprotic solvents such as chloroform, acetonitrile and acetone are centered in the range of 494–538 nm. In the presence of alcohols and DMF as the potential hydrogen bond donors, the fluorescence spectra shift towards 536–560 nm [69].

**Fig. (2):**
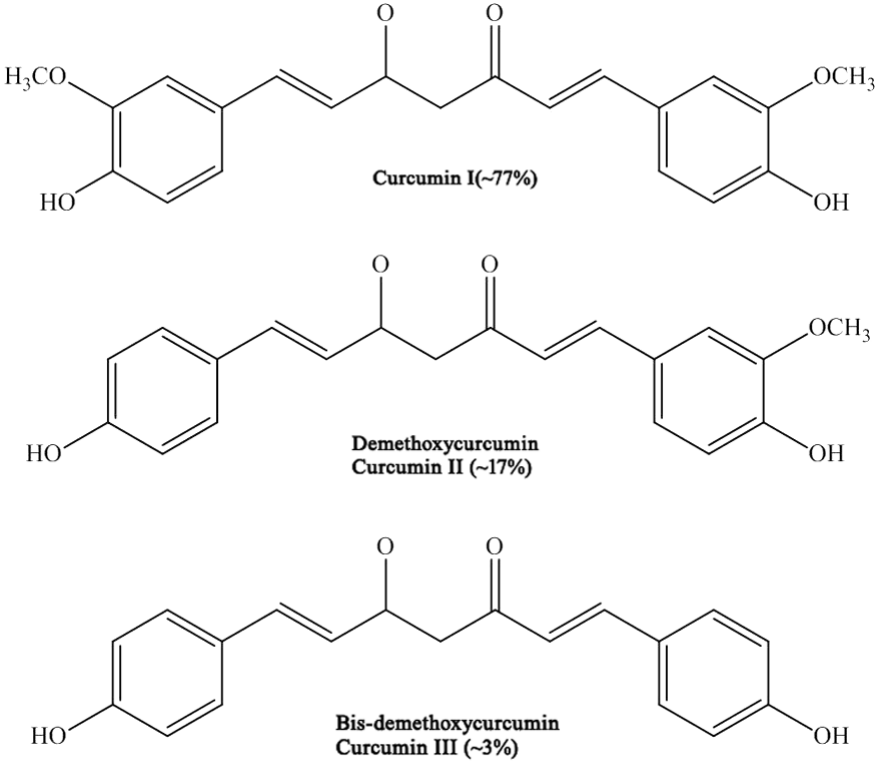
Chemical structure of curcumin, demethoxycurcumin and bis-demethoxycurcumin.

Curcuminoids complex, found in the rhizome of turmeric (2.5–6%) comprises curcumin (curcumin I), demethoxycurcumin [4-hydroxy-cinna [pmoyl- (4-hydroxy-3-methoxycinnamoyl)methane] (curcumin II), bisdemethoxycurcumin [bis-ane] (curcumin III) and cyclocurcumin (Fig. **2**). The physicochemical properties of major curcuminoids are listed in Table **2**. In commercial grades of curcumin, the major curcuminoid complexes present are curcumin I (77%), curcumin II (17%) and curcumin III (3%). In addition, three minor constituents believed to be geometric isomers of curcumin have also been isolated. Identification of one of these compounds is leaning towards *cis-trans* geometrical configuration based on its comparative lower melting point, stability and maximal UV absorption intensity to the trans-trans configuration.

### Stability of Curcumin in Aqueous Solution

Stability of curcumin is crucial to maintain its physiological activities. In acidic and neutral solutions (pH 2.5–7.0), curcumin emits a bright yellow hue while it turns red at pH above 7 [70]. The decomposition of curcumin is pH-dependent and it degrades more rapidly at neutral-basic conditions. In acidic conditions, degradation of curcumin is slow, with <20% of curcumin decomposed after 1 h. Curcumin undergoes rapid hydrolytic degradation at physiological pH or greater which becomes a significant disadvantage in its therapeutic use [60, 71]. 

**Fig. (3):**
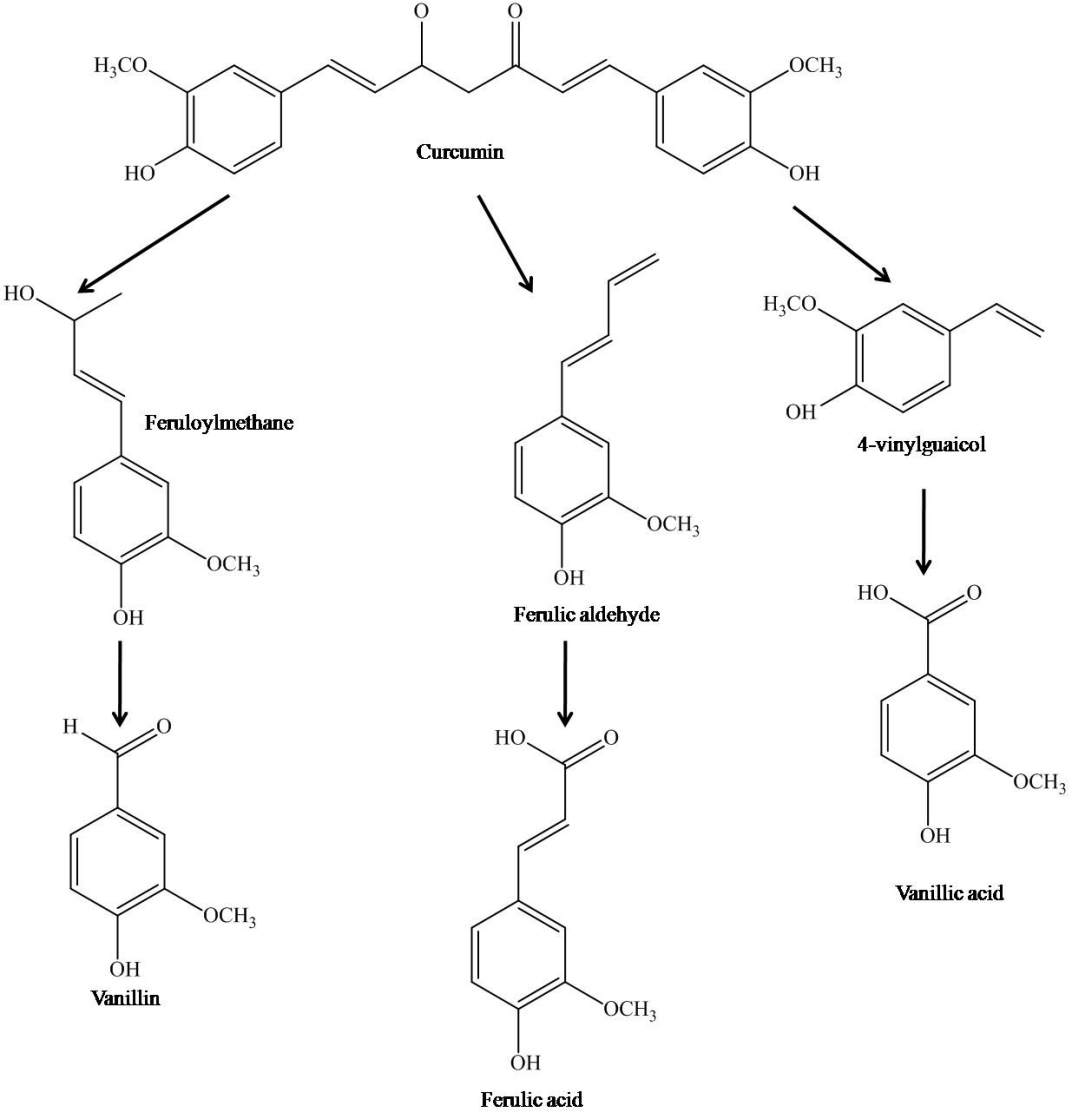
Degradation products of curcumin under physiological conditions.

Studies on curcumin’s stability towards hydrolytic degradation in alkaline solutions presented varying results, possibly due to differences in the media used [60, 62, 72, 73]. Tønnesen *et al.* observed that the degradation of curcumin followed second-order kinetics in methanol/aqueous system with either phosphate (pH 6–9) or carbonate (pH 9–10) as buffers and it was concluded that the degradative reactions of curcumin proceeded at a much slower rate in acidic conditions than at higher pH [73]. In a methanol/aqueous system, ferulic acid and feruloylmethane were the initial degradation products, followed by hydrolysis of feruloylmethane into vanillin and acetone (Fig. **3**) [73]. In addition, a brownish-yellow like compound was also present, believed to be a product from the condensation of feruloylmethane [73].

In another study by Wang *et al*., more than 90% of the curcumin decomposition in 30 min was noted when incubated in a serum free 0.1 M phosphate buffer (pH 7.2) at 37 °C. The degraded products were identified as trans-6-(4’-hydroxy-3’-methoxyphenyl)-2,4,-dioxo-5-hexenal (major products), vanillin, ferulic acid and feruloylmethane using high performance liquid chromatography (HPLC) and gas chromatography mass spectrophotometry (GC-MS) analyses. The presence of 10% fetal calf serum in cell culture medium and human blood improved the hydrolytic stability of curcumin, whereby approximately 20% was degraded after 1 h of incubation and the degradation half life of curcumin was increased to 8 h compared to the 90% decomposition rate in 30 min in a free-serum culture medium [60]. Addition of anti-oxidants such as ascorbic acid, *N*-acetylcysteine and glutathione slowed the degradation of curcumin in both cell culture medium and phosphate buffer under alkaline conditions by decelerating the oxidation of curcumin [60]. The complexation of curcumin with cyclodextrin improved its solubility to more than 10 000 folds in aqueous solution at pH 5 [74, 75]. 

**Fig. (4):**
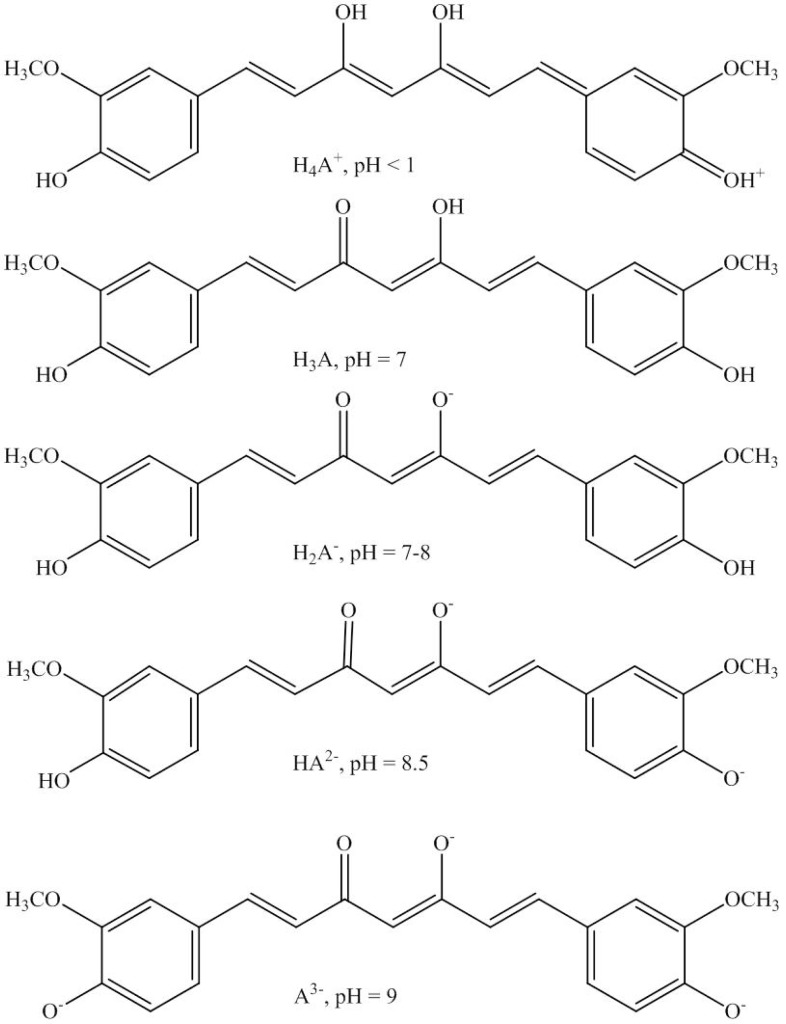
The kinetic behavior of curcumin degradation in various pH systems. At pH < 1, curcumin appears in protonated form (H4A+). In a solution of pH 1–7, curcumin exists in the neutral form (H3A). In the pH range of 8.08 to 8.75, it is postulated that curcumin exists in equilibrium in three forms: H3A, H2A and HA2.

The kinetic behavior of curcumin degradation in various pH systems is complex as demonstrated in the study by Tønnesen and Karlsen [71]. Curcumin has a half-life of approximately 6.6 x 10^3^ h at pH 1.23, but is shortened as the pH is increased to 7.98. Between pH 8.08 to 8.75, minimal curcumin degradation is observed but increases rapidly at pH > 9. The peaks and valley in the pH regime hint at the possible involvement of acid-base equilibrium during degradation. As illustrated in Fig. (**4**), at pH < 1, curcumin is red and appears in the protonated form (H_4_A^+^). In a solution of pH 1–7, curcumin is yellowish and mostly exists in the neutral form (H_3_A). Curcumin is more stable in acidic conditions probably owing to its conjugated diene structure which is gradually destroyed as the proton is removed during dissociation of phenolic groups in the curcumin structure (H_2_A^−^, HA^2−^ and A^3−^) at higher pH, thus causing curcumin to be more prone to degradation as detected using HPLC (Fig. **4**). In the pH range of 8.08 to 8.75, it is postulated that curcumin exists in equilibrium in three forms: H_3_A, H_2_A^−^ and HA^2−^ [71].

### Photodegradation of Curcumin

Curcumin is also sensitive to light and is rapidly decolorized upon exposure to UV light [76, 77]. According to Auslander *et al*., complete color loss of curcuminoid pigments in turmeric occured within three days of exposure to 900 footcandle of visible light [78]. Khurana and Ho reported that the dried powder form of curcumin was more stable to photo-oxidation as compared to the alcoholic extract [77]. In addition to that, the rate of curcumin photodegradation was influenced to a large extent by the nature of solvents used [77]. Tønnesen and Karlsen showed that stability of curcumin (as measured by half-life in various solvents) followed the decreasing trend: methanol (92.7 h) > ethyl acetate (15.1 h) > acetonitrile (6.3 h) > chloroform (2.7 h) [71]. It was probable that the significant protective or destabilizing effect of different solvents could be ascribed to the formation of inter- and intra-molecular bondings, which were solvent dependent [71]. Souza *et al.* found that the combination effect of air and light was more detrimental than light alone while the absorption of curcumin in water had no apparent effect on curcumin photo-stability [79]. In another study, the order of photochemical stability of different curcuminoid complexes in air-sparged conditions in methanol was curcumin > demethoxycurcumin > bis demethoxycurcumin [80]. 

Although the mechanism of photodegradation is yet to be elucidated, it is evident that the presence of phenolic hydroxyl groups or lack of them does not play any distinctive role in curcumin photodegradation. Instead, it is more likely that the β-diketone moiety is involved in scavenging of the hydroxyl radical and redox reactions, thus forming smaller phenolic compounds. Under the influence of light, curcumin acted as photo-sensitizer of oxygen radicals and underwent self-sensitized decomposition [76]. A yellow cyclization compound formed by the loss of two hydrogen atoms of curcumin molecule was the main degradation product under visible light (λ > 400 nm). According to Tønnesen *et al*., ferulic aldehyde, ferulic acid, 4-vinylguaiacol, vanillin and vanilic acid were formed as a consequence of various photochemical reactions between curcumin and oxygen radicals (Fig. **3**) [76, 81]. In addition, other byproducts such as benzaldehyde, cinnamaldehyde, 2'-hydroxy-5',6'-benzochalcone, flavanone and unknown photoproducts were also identified from the photodegradation of non-phenolic curcuminoids [82].

### Phototoxicity of Curcumin

Curcumin is known to exert direct antibacterial activity against a wide spectrum of bacteria [83-88]. In the absence of light, curcumin exhibited negligible effect against bacteria [83]. Various studies had compared the killing efficacy of curcumin between Gram positive (*Staphylococcus aureus*, *S. intermedius*, *Enterococcus faecalis*) and Gram negative bacteria (*Escherichia coli*,* Salmonella typhimurium*, *Sarcina lutea*) (Table **3**). 

Tønnesen *et al.* studied the photobiological effect of curcumin on bacterial systems and demonstrated that under visible light, curcumin possesses phototoxic effects on *Salmonella typhimurium *and *E. coli* at very low concentrations [83]. This was followed by the work by Dahl *et al*., who evaluated the phototoxicity of curcumin in bacterial systems and their results showed that Gram-negative bacteria possessed greater resistance to curcumin phototoxicity compared to Gram-positive bacteria [89]. The authors also examined the phototoxicity of curcumin in mammalian cells (rat basophilic leukemia cells) and demonstrated that the death profile of this cancer followed a first order kinetic [89]. Recently, the phototoxic effects of curcumin were evaluated with salivary gland acinar cells and remarkable results were obtained in which few micromolar concentrations of curcumin coupled with low light illumination were effective in the treatment of oral lesions and cancers [90]. Curcumin could potentially act as photosensitizer and chemotherapeutic agent by causing cell shrinkage, membrane bleeding and apoptosis. The photosensitization characteristics of curcumin could be employed as a phototherapeutic agent for cancer treatment, as well as in microbial diseases. 

### ALZHEIMER’S DISEASE (AD) PATHOGENESIS

AD, discovered in 1907 by a German medical researcher Dr. Alois Alzheimer, is a progressive neurodegenerative disorder of the central nervous system (CNS) occurring commonly in the elderly population. Statistics revealed that one out of ten individuals over 65 years old and approximately 50% of individuals above 85 years old are affected with AD [111]. Early onset AD is associated with subtly impaired cognitive function or impaired judgment, which gradually develops into memory dysfunction, loss of lexical access, spatial and temporal disorientation. Pathological changes associated with AD include the presence of extracellular senile plaques mainly comprised of A*β* peptides, intracellular neurofibrillary tangles characterized by aggregates of microtubule-associated protein, oxidative damage, inflammation, impaired cognitive function and neuron loss [33, 112-116]. 

## FACTORS CONTRIBUTING TO AD 

### Key Molecules Involved in AD Pathogenesis 

The contributing factors to AD involve both genetic and non-genetic components. Non-genetic factors that contribute to the prevalence of AD are closely affiliated to lifestyle and other factors including diabetes, advanced age, obesity, trauma and cardiovascular diseases. The genetic factors contributing to AD are complex, heterogeneous and involve mutations and polymorphisms of several genes. Mutations of amyloid β precursor protein (APP) and presenilin (PS 1 and 2) cause autosomal dominant AD, which manifest as early-onset AD pathogenesis [117]. Mutations of these three genes are associated with alterations on three different chromosomes: APP on chromosome 21, PS 1 on chromosome 14 and PS 2 on chromosome 1 [118-120]. Although these mutations are present in different chromosomal locations, the altered genes trigger the same biochemical pathway notably in the altered expression of A*β* peptides that lead to neuronal death and therefore AD. For instance, a common mutation in APP known as the Artic mutation increases A*β* deposition while the mutation of PS1M146V PS results in elevated levels of readily aggregating A*β*_42 _[121-123]. 

APP is a mammalian transmembrane protein that contains 695–770 amino acid residues with a large ectocytoplasmic N-terminus domain and a cytoplasmic carboxyl-terminal region [124]. APP is a precursor for the synthesis of A*β *that undergoes endoproteolysis either through non-amyloidogenic or amyloidogenic pathways achieved by sequential cleavage by a complex of enzymes known as α-, β- and γ- secretases [125]. In the prevalent non-amyloidogenic pathway, APP is cleaved by the membrane-bound enzyme α-secretase [125] within its A*β *domain, resulting in the formation of extracellular secretion of neuroprotective soluble APPα (sAPPα) fragments [126] and short a membrane-bound C-terminal fragment (CTF or α-CTF or C83) followed by γ-secretase cleavage of C83 to form a 3 kDa peptide (p3) with release of the APP intracellular domain (AICD) into the cytoplasm [127, 128]. On the other hand, in the amyloidogenic pathway, APP at the N-terminal part of the A*β* domain is cleaved by β-site APP cleaving enzyme 1 (formerly known as β-secretase) (BACE-1), resulting in release of extracellular soluble fragments of sAPP*β* and a membrane bound C99 or β-CTF [129, 130]. subsequent cleavage of C99, which critically requires the γ-secretase enzyme in particular PS, at the C-terminal of A*β* produces A*β* peptides with different lengths and numbers of amino acids residues – the etiological agent for Alzheimer pathology [127]. γ-secretase is complex of enzymes composed of PS1, PS2, nicastrin, anterior pharynx defective and PS enhancer 2 [118, 128, 131, 132]. BACE-1 is a 75 kDa protein, synthesized in endoplasmic reticulum and transported to Golgi network as pre-BACE-1 that has the initial approximate molecular weight of 65 kDa [124]. It subsequently forms active BACE-1 after undergoing maturation and post-translational modifications. It should be noted that besides BACE-1, a close homolog BACE-2 is also identified but there is no clear evidence relating any cleavage activities of this secretase in human APP protein. While the expression of BACE-1 is high in neurons, BACE-2 is most commonly found in peripheral tissues such as the pancreas and stomach [133]. BACE-1 appeares to be the active enzyme in amyloidogenesis in brain [134, 135]. Antisense inhibition of BACE-1 rather than BACE-2 reduces the formation of A*β *[136, 137]. 

Apolipoprotein E (ApoE) is a plasma glycoprotein mainly synthesized in liver, neurons and astrocytes, macrophages and monocytes. This 34 kDa protein is responsible for the redistribution of lipids in the brain through the low-density lipoprotein and related receptors [138]. Three major isoforms of ApoE, identified using isoelectric focusing are tentatively named Apo2, Apo3 and Apo4 [139]. These alleles differ in their amino acid sequences at residues 112 and 158 whereby Apo2 contains cysteine residues, Apo3 contains cysteine and arginine, while Apo4 contains arginine at both sites [140]. Although the anti-oxidant property of Apo2 appears to protect against the development of AD possibly *via *minimizing A*β *aggregation, patients carrying Apo4 alleles show increased deposition of A*β *[141]. This is in accord with statistics which show that the ApoE frequency in AD patients is increased three-fold with Apo4 being the most abundant form of the allele at 45%. Apart from the low anti-oxidant trait, Apo4 also possesses hyperinflammatory behaviour, which ultimately contributes to brain damage in AD patients [142]. 

### Mechanisms (Aβ Aggregation and Tau Hyperphos-phorylation)

**Fig. (5):**
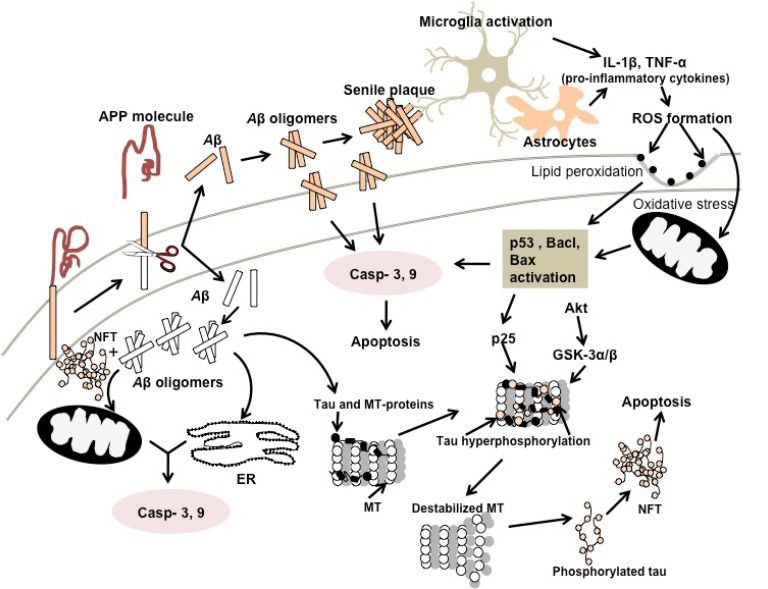
AD is characterized by the presence of extracellular senile plaques mainly comprised of Aβ peptides, NFT associated with aggregates of tau, oxidative damage, inflammation, impaired cognitive function and neuron loss. In the disease state, APP is sequentially cleaved by secretases resulting in the formation of both extra- and intra- cellular Aβ oligomers which eventually aggregates and forms senile plaques. The neurotoxic Aβ oligomers cause impaired mitochondrial functions, lipid peroxidation, oxidative stress related damages and activations of pro-inflammatory cytokines, p53, Bacl, Bax and caspases. The presence of NFT is also a central hallmark of AD pathogenesis. NFT is formed as a result of hyperphosphorylation of tau leading to destabilization of microtubule (MT) and aggregation of tau protein. This cascade of event eventually leads to apoptosis of neurons.

In the mid 1980s, A*β* peptides are considered as extracellular amyloid plaques. The β-sheet organization of A*β*_10–35_, was first studied using solid state magnetic resonance spectroscopy and subsequent studies revealed that the parallel full length A*β*_1–42 _form of β-sheets had the same orientation [143, 144]. Newly produced, cell secreted soluble A*β*, which assume random β-helix conformations, is commonly referred to as A*β* monomer [145]. A*β *monomer readily aggregates to form several complexes ranging from low molecular weight dimers to high molecular weight fibrils (Fig. **5**). In general, oligomeric A*β *is a common term used to describe peptide aggregates with limited stoichiometry such as dimers and trimers**. **A spheroidal structure referred to as A*β *protofibrils (PF) is an intermediate structure between fibrils and aggregates [146]. Meanwhile, A*β*-derived diffusible ligands (ADDLs) are defined as intermediates to protofibrils. Fibrils, the final assembly form of A*β* are the basic component for amyloid plaques formation [145]. 

A*β *is a non-toxic soluble monomer present plasma and cerebrospinal fluid and is also secreted by cells in culture [128, 147-149]. The presence of amyloid plaques in essentially most AD patients lead many researchers to firmly believe that the conversion of soluble A*β *into insoluble fibrils is critical for the onset development of AD pathogenesis [150]. The high toxicity of aged A*β *(comprised of insoluble amyloid fibrils) compared to fresh A*β *monomer is in accordance with this hypothesis. However, recent evidence suggests that the absolute requirement of the fibril form of A*β* in early AD pathogenesis is not always the case as the deposition of amyloid fibril is often unparallel to the incidence of dementia [151, 152]. In addition, these amyloid fibrils are also commonly found in the brain of healthy individuals. Studies have demonstrated that soluble aggregates of A*β *including oligomers, ADDLS and PF are toxic and pathological states of AD with oligomer species exerting the highest damage, from dimers disrupting memory and synaptic functions to dodecamers causing cognition failure [153-155]. 

Earlier studies had generally described A*β* as a component of extracellular amyloid plaques. Grundke-Iqbal *et al.* demonstrated for the first time the presence of A*β *intraneuronally, in which the antibody against residues 17–24 of A*β* had successfully detected traces of A*β*-immuno-reactive materials within the cerebellum, cerebrum and spinal cords of subjects with or without AD pathology [156]. Several studies on post mortem AD and Down syndrome transgenic mouse brains revealed the accumulation of A*β* within neuron, which provided support for the earlier observation [157-159]. This indicates that the build up of A*β *intraneuronally is an indicator of early events in AD pathogenesis. Patients with mild cognitive impairment (MCI) were reported to have accumulations of intracellular A*β*-immunoreactivity, which were prone to initiate early AD pathology, subsequently preceding the formation and deposition of extracellular A*β *plaques [157, 158]. The fact that A*β *is produced in the body throughout life suggests that A*β *serves a physiological role. With old age, specifically with the onset of AD pathology, it is believed that A*β *had either lost its physiological capacity or gained pathological traits or maybe both. This notion is supported by the structural changes of soluble A*β *monomers with age from its random coil or β-helix conformation into a β-sheet which further leads to the formation of low-molecular weight oligomers, higher molecular weight complexes such as PF and ADDLs. A*β* peptides consisting less than 41 amino acid residues, (A*β*_1–40_) is the most prevalent form and less pathogenic in terms of AD development. However, although A*β* with 42 amino acid residues (A*β*_(1-42)_) is available in low quantities, it is highly prone to aggregation and readily self-assembles to form a heterogenous mixture of oligomers and PF subsequently deposited as fibrils in senile plaques [160, 161]. 

As mentioned above, soluble oligomeric forms of A*β *are more neurotoxic than other assembly states of A*β*. Walsh *et al*. reported that A*β* oligomers were formed initially within brain cells rather than in the extracellular region [162]. Intracellular A*β *in the brain possibly served as a ‘seed’ mediating the formation of toxic oligomers, which then in turn were secreted to the extracellular environment and further implicated in AD pathogenesis. The interaction of A*β* with clusters of brain gangliosides resulted in localization of ganglioside bound A*β* on cell surfaces, which functioned as a nucleation site for internal A*β* oligomerization [163]. In addition, studies on brain ganglioside derived from circular dichroism spectroscopy and electron microscopy suggested that these interactions caused the changes in A*β *peptide structural folding as gangliosides induce a novel conformation in A*β*_40/42_ and promote β-sheet structures [164, 165]. These findings revealed that lipid bilayers containing ganglioside served as a chaperone or reservoir by providing a site for membrane bound active A*β* with seeding ability and the presence of intracellular membranes further elevated the generations of toxic oligomers. On the other hand, acidic phospholipids also interacted with A*β *by signalling peptide accumulation and accelerating A*β* unfolding into β-sheet and fibrillar formation [166]. The accumulation of A*β* within neuron cells also disrupted the activity of the ubiquitin-proteasome [167, 168]. Impaired proteasome has been known to lead to an increase in tau protein levels [167, 168]. 

Tau protein is highly soluble as it mostly contains hydrophilic residues and exists in a natively unfolded state under physiological conditions. This protein is a neuronal microtubule-associated protein and functions primarily to stabilize microtubules (MT) through interactions with tubulin and subsequent incorporation into MT assembly, as it is found in abundance in the axons of neurons [169] (Fig. **5**). Additional roles of tau include the regulation of MT-dependent axonal transport through its binding to motor protein kinesin and prevents neurons from acute apoptosis [170]. In a healthy adult human brain, tau exists in six different isoforms, which are generated through alternative splicing of the tau gene located at chromosome 17 [171]. Under the influence of pathological conditions, phosphorylated tau dissociates from MT, increasing the levels of unbound tau, and subsequently enhances fibrillization and aggregations into neurofibrillary tangles (NFT) [112, 172] (Fig. **5**). The loss of MT stabilization effect by tau compromises regulatory functions of cytoskeleton, axonal transport and eventually leading to neuronal malfunction, neurodegeneration and AD [173]. The condition is exacerbated by the accumulation of relative large and ‘bulky’ NFT that might indirectly disrupt the cellular functions and sequestration activity of tau and other proteins and further deteriorates the physiological functions of tau. Hyperphosphorylation of tau is believed to be the hallmark of aggregation and NFT formation and AD pathogenesis. In healthy brains, only 2 to 3 residues of tau are phosphorylated while the phosphorylated tau level in AD patients is significantly higher at 9 phosphates per molecule of tau [174, 175]. Hyperphosphorylated tau has lower binding affinity towards MT and is generally more resistant to degradation by proteases and ubiquitin-proteosome pathways [176]. 

Several pathogenic events have been suggested to either contribute directly or indirectly to abnormal phosphorylation of tau. The mutation of chromosome 17 linked to frontotemporal dementia (FTD) with Parkinsonism showed a direct correlation with the occurrence of tau hyper-phosphorylation. Pathologically, all FTD-17 cases showed the presence of filamentous fibrils of phosphorylated tau. Thus, genetic mutations resulted in expression of tau mutants that possessed the characteristics such as: a) easily phosphorylated, b) impaired MT binding and c) deposited as filamentous fibrils which were more prone to form NFT [118, 137, 177-181]. The imbalance in the activity of kinase and phosphatase enzymes that is responsible for phosphorylation and dephosphorylation is another factor contributing to tau malfunction. Both enzymes are required in normal flux for MT stability and effective axonal transport. The kinase enzymes that are implicated in hyperphosphorylation include glycogen synthase kinase 3β (GSK-3β), cyclin-dependent protein kinase 5 (CDK5), cAMP-dependent protein kinase (PKA), mitogen-activated protein kinase (MAPK), calcium-calmodulin-dependent kinase-II (CAMK II) and microtubule affinity-regulating kinase (MARK) [182-186]. Among them, GSK-3β and MARK have received significant attention as potential therapeutic targets for AD treatment using kinase inhibitory drugs. Similarly since protein phosphatases such as PP1, PP2, PP2B and PP2C have been shown to reverse the phosphorylation activities, approaches to prevent NFT formation in AD has been focused to increase phosphatase activity [187]. Other upstream events such as oxidative damage, inflammation and A*β*-mediated toxicity could possibly trigger the detachment of tau from MT. Recent studies had demonstrated that the transcripts of pro-inflammatory markers in AD were elevated as a response to tau, thus establishing the direct relationship between neuro-inflammation to tau hyperphosphorylation and AD pathogenesis. In addition, oxidative stress involved several covalent modifications in tau such as disulphide bridge formation, which resulted in tau misfolding, aggregation, hyperphosphorylation and subsequent removal of tau from MT [188-190]. Although the notion of tau toxicity and pathology mediated by A*β* has been suggested, the exact mechanisms that link both components are yet to be fully understood.

### Oxidative Damage to Lipid, Protein and Nucleic Acids in Brain. What is the Relationship between Aβ-mediated Toxicity and Oxidative Stress?

Oxidative stress is characterized by the overproduction of ROS, O_2_^-^ and H_2_O_2_ that arises as a result of imbalanced equilibrium between pro-oxidant and anti-oxidant homeostasis. In healthy brains, anti-oxidants are present in abundance to counteract the ROS generated from redox-active metal-ion reduction and activation of molecular oxygen *via *Fenton chemistry. ROS can be dangerous to cells as well as human organs since they can attack lipids of cell membranes, enzymes, carbohydrates and DNA, leading to oxidative process. The human body is equipped with sophisticated anti-oxidant systems to protect against the deleterious effects of free radicals generated from ROS. These systems include a) enzymatic defences such as catalase, superoxide dismutase and glutathione that inhibit the formation of toxic OH^-^ by metabolizing superoxide, H_2_O_2_ and lipid peroxides, and b) entrapping free radical defences such as histidine-peptides, the iron-binding proteins transferring and ferritin, dihydrolipoic acid, reduced CoQ_10_, melatonin, urate and plasma protein thiols [24, 191-193]. 

The role of oxidative stress in AD pathogenesis is well established with substantial evidence demonstrating evidence of ROS-mediated injury and free radical damage to key intracellular targets such as DNA or mitochondria in AD brains (Fig. **5**). The neurons are particularly susceptible to oxidative damage based on the following: a) high content of readily oxidised polyunsaturated fatty acids in the membrane, b) low levels of natural anti-oxidant component (i.e. glutathione and vitamin E) and anti-oxidant enzymes (glutathione peroxidase, catalase and superoxide dismutase), c) the high consumption of oxygen required for brain metabolism, d) high content of lipid peroxidation metals (i.e. iron and ascorbate), and e) inability of neuronal cells to replicate. In AD, the overproduction of free radicals is most probably related to mitochondrial anomalies, lipid peroxidation, A*β *peptides and ApoE and the presence of unbound metal ions (Fig. **5**). It is generally assumed that mitochondria anomalies trigger the production of uncontrollable free radicals and slow down energy metabolism. The oxidation of mitochondrial DNA is evidently higher in the frontal, parietal and temporal lobes in AD brains compared to control subjects [194]. Wang *et al.* showed that mitochondrial DNA suffered more oxidative insult than nuclear DNA as seen in the 10-folds difference of the oxidised bases levels between two sites [194]. In addition, temporal lobe is the most susceptible site for oxidative injury while damage to cerebellum is almost negligible. ROS is known to cause modification of purine and pyridine bases of DNA and RNA. The levels of 8-hydroxyguanine, the marker for RNA oxidation, were unusually high in AD and MCI patients [195]. In the mitochondria, as the electron transport chains are involved in the formation of ATP *via *reduction of oxygen to water, it is assumed that any defect in these chains could contribute to free radical formations. Several studies had demonstrated that the activity of cytochrome *c* oxidase (part of a complex enzymatic system in the mitochondria) was significantly lower in AD than normal [196-198]. At least 25% loss of cytochrome *c* oxidase activity was observed in the cerebral cortex [198] while in another study, similar observation was recorded in dentate gyrus and CA4, CA3 and CA1 of the hippocampus [199]. How does the affected cytochrome *c* oxidase activity correlate to the development of AD? Lakis *et al.* confirmed that the alteration of this enzyme’s activity led to the overproduction of damaging free radicals and eventually oxidative damage to neuron [200]. The slowing down in energy metabolism in pyruvate dehydrogenase and oxoglutarate dehydrogenase is another common trait found in AD pathogenesis. An *in vitro* study using AD fibroblasts revealed a reduction of oxoglutarate dehydrogenase activity and it was further supported by an *in vivo* study with AD patients whereby the activity of oxoglutarate dehydrogenase complex was markedly reduced by 46 to 68% [201, 202]. 

The attack of ROS on membrane phospholipids known as lipid peroxidation caused alterations and depletions in the lipids that could be specific to the pathogenesis of AD. Free 4-hydroxynoneal (4HNE) and malondyaldehyde, the signs of lipid peroxidation, are present at higher levels in several regions of AD brain and cerebrospinal fluid in which the former is toxic to hippocampal cell cultures at high concentrations. This aldehyde is precursor to neuron cell death as the modification of ATPase activity in ionic transfers and calcium homeostasis from their association causes an increase in ROS and subsequent cascade of intracellular events. 4HNE is also believed to be more harmful than ROS due to its longer half-lives. In addition, F_2_-isoprostanes, a product from ROS-induced arachidonic oxidation is elevated in AD brain regions. ROS is also a promiscuous pro-oxidant as their association with proteins resulting in protein carbonyl and 3-nitrotyrosine (3-NT) is evidently higher in AD patients. 

Although the exact mechanism surrounding A*β*-mediated toxicity is only partially understood, it is strongly possible that oxidative stress is closely involved in the initiation of its toxicity. As reported by Behl *et al*. the presence of anti-oxidants such as vitamin E alleviated the A*β*-mediated toxicity induced by H_2_O_2_ on PC12 cell lines [203]. Concurrently, high amount of enzyme catalase, which was responsible in protecting cells from the toxic effect of A*β *by degrading H_2_O_2_, was expressed in PC12 [203]. In line with this, Mark *et al*. reported significant increase in 4HNE levels when hippocampal neurons were exposed to A*β* [204]. The matter is further complicated with the discovery that A*β* is not only a victim of oxidative stress, A*β *and their complexes are also a source of ROS. Activation of nicotinamide adenine dinucleotide phosphate (NADPH) by A*β *led to the production of ROS, causing direct toxicity to neuron. Furthermore, A*β* is identified as an indirect culprit in lipid peroxidation by interacting promiscuously with endothelial cells that produces ROS and lipid oxidising agents. 

### Accumulation of Metal Ions in Brain and Metallo-biology in AD

Metal ion homeostasis in brain is tightly regulated to maintain its physiological processes. Recent evidence showed that imbalances of metal ions (Fe^3+^, Cu^2+^, Zn^2+^ and Al^3+^) homeostasis had implicated them with A*β* aggregation and subsequent induction of oxidative damage. The presence of Fe^3+^ and Cu^2+^ increased the A*β *toxicity while Zn^2+^ attenuated it [205]. Post mortem analysis of AD patients revealed unusually high levels of these metal ions in the senile plaques [Cu^2+^ (393 μM), Zn^2+^ (1055μM) and Fe^3+^ (940μM)] compared to a healthy person [Cu^2+^ (70 μM), Zn^2+^ (350μM) and Fe^3+^ (340μM)] [206, 207]. The high affinity of A*β* Fe^3+^, Cu^2+^ and Zn^2+^, caused its aggregation and oligomerization. The identified metal binding sites in A*β* are three histidine (His13, His14 and His 6) and one tyrosine (Tyr10) residues located at hydrophilic N terminus regions [208-210]. 

Fe^3+^ has been found in the senile plaques and NFT of AD patients where it is abundantly localized in neocortical grey matter, amygdale and olfactory tract [211-213]. The contribution of Fe^3+ ^to neuronal toxicity involves facilitating both A*β *and tau aggregation and upregulating the APP translation, which in turn produces more A*β *[214, 215]. Fe^3+^ is known to stimulate free radical formation particularly converting H_2_O_2 _to highly reactive and toxic OH^- ^through Fenton reaction, in which the presence of either compounds are malicious and a threat to initiate oxidative damage as stressed in the earlier section of the review [216]. It is established that Fe is more reactive in its Fe^2+^ form compared to its Fe^3+^ form. Since A*β* has high positive reduction potential, it rapidly reduces Fe^3+^ into Fe^2+^ and thereby generates oxidised A*β* radicals while Fe^2+^ reacts with oxygen to form H_2_O_2_. In addition, Fe^3+ ^tends to accumulate in neurons together with NFT as Fe^3+^ binds to different motif of hyperphosphorylated tau, resulting in altered conformation, aggregation and subsequent NFT formation [217]. 

Similar to Fe^3+^, Cu^2+ ^binds to A*β* and elicits similar response in terms of activation of A*β *aggregation, H_2_O_2_ generation and other oxidative stress-mediated neuronal toxicity [215, 218, 219]. Specifically, Cu^2+^ has high binding affinity towards A*β* at His13, His14, His6 and Tyr10 residues [220]. The neurotoxicity of Cu^2+ ^was dependent on the chain length of A*β* peptide whereby the toxic response were elevated for the peptide with longer amino acid residues (A*β*_1–42_) probably attributable to a bigger capacity to reduce Cu^2+^ owing to its higher positive reduction potential [205]. It is reported that Cu^2+ ^has synergistic effect with A*β* in the inhibition of cytochrome *c *oxidase complex of the mitochondrial electron transport chain. The A*β*/Cu complex required ~0.75 mole metal per mole of peptide for successful inhibition of the enzyme’s activities, thus suggesting the formation of a reactive by-product (from the complexation) that reacted to cytochrome *c *oxidase [221-223]. In normal physiological conditions, the release of substantial amount of Cu^2+^ (up to 15 μM) into the synaptic space makes the ions susceptible to binding to extracellular A*β*, which accelerates oligomerization and senile plaques formation. However, contradictory results which showed a deficiency of Cu^2+^ in the development of AD are intriguing. Evidence supporting the deficiency of Cu^2+^ in relation to AD is clearly seen in the inverse relationship between the Cu^2+ ^concentration and A*β* found in cerebrospinal fluid of AD patient [224]. 

On the other hand, Zn^2+ ^displayed neuroprotective characteristics as evident from its inhibition of A*β*-mediated toxicity in the presence of low concentrations of Zn^2+^ (200–300 μM) [225]. As intriguing as it may seem, it appeared that the possible explanations for the neuroprotective trait conferred by Zn^2+^ could be the blockage of membrane calcium pore channel formed by A*β*_1–40_ and enhancement of Na^+^/K^+^ ATPase [226-229]_. _Futhermore, Zn^2+^ is involved in an inhibitory competition with Cu^2+ ^(or Fe^3+^) for the binding to A*β *[205, 230]. The binding of Zn^2+^ to A*β *altered the peptides’ structural conformation, inhibited the interaction between Cu^2+^ and the binding sites of A*β*, and therefore indirectly reduced the toxicity induced from A*β*/Cu complex. However at high concentration, the binding Zn^2+ ^to A*β* formed toxic A*β *aggregates and further cascade of events leading to A*β*-mediated toxicity neuronal death [231]. An interesting therapy for AD could be focused on metal chelator drugs or compounds since the above-mentioned metal ions are involved in the free radical generations and subsequently imposing oxidative damage. The metal-chelating properties of curcumin will be discussed in later in this review. 

### Neuroinflammation

Inflammation is a complex cellular response to stress, injury and infection where the depositions of insoluble materials in the periphery are the classic trigger for inflammation. Likewise, in the neuron, the presence of insoluble senile plaques, A*β* and NFT is the stimulant for inflammatory response. The neuroinflammation is characterized by the activation of microglia, astrocytes, macrophages and lymphocytes, which over express mediators such as cytokines, chemokines, prostaglandins, acute proteins, neurotransmitters and ROS/RNS [232, 233]. 

Microglia cells are the first line of active immune defense in CNS, which is activated in response to pathological lesions such as A*β* and plaques. Microglia cells respond by clustering themselves around A*β *deposits while secreting scavenger receptors to aid the cells adhesion onto A*β* surfaces. Activated microglia cells by A*β *showed an increase in the secretion of pro-inflammatory cytokines (interleukin-1β) IL-1β, IL-6 and (tumor necrosis factor-α) TNF-α, chemokines (IL-8), ROS/RNS and the complement protein/inhibitors [234-236]. Secretion of chemokines leads to the increase of BBB permeability, enhancing transport of monocytes to CNS, and promoting flux of microglia cells [237]. In aging brain, the signals mediated by microglia cells do not induce neuroprotective homeostasis responsive, but rather evoke exaggerated and continuous flux of microglia and subsequent toxic compound (i.e. RNS and ROS) that elicit AD pathology progression. Independent studies in AD patients by Forlenza *et al*. and Holmes *et al*. provided direct evidence that increased plasma levels of IL-1β correlated with higher cognitive decline, thus suggesting the possible progression of AD from systemic inflammatory events [238, 239].

Astrocytes, a star-shaped glial cell, are involved in regulating the permeability of BBB, transporting of nutrient cells to CNS and repairing mechanisms of brain and spinal cord induced by trauma injury [233]. Particularly in their response to A*β *plaques deposition in CNS, the crucial task of astrocytes is in A*β *clearance and degradation [240]. Similar to the response evoked by microglia, astrocytes activated by A*β* produced various pro-inflammatory compounds such as IL-1, IL-6, IL-18, TNF-α, prostaglandins and ROS [241, 242]. AD brains showed high levels of nitrous oxide synthase astrocytes secretion compared to control while the over expressed cytokine S100B triggers the AD-like pathology in Tg2576 mouse model [243, 244]. In addition, chemokines secreted by astrocytes work synergistically with microglia, which further express pro-inflammatory products, thus increasing the risk of neuronal damage in AD. 

## NEUROPROTECTIVE ACTION OF CURCUMIN AGAINST AD 

### *In Vitro *Evidences 

**Fig. (6):**
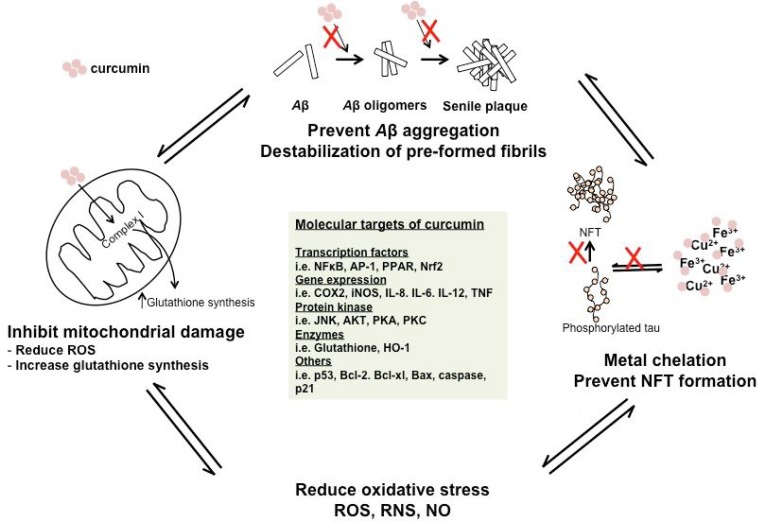
Proposed molecular targets of curcumin against AD. Curcumin is known to prevent Aβ aggregation and destabilizes pre-formed fibrils. In addition, curcumin protects from mitochondrial dysfunction by decreasing the load of ROS and increases the synthesis of glutathione. Curcumin also prevents neuronal loss from oxidative damage by scavenging NO-based radicals; which thus neutralizes ROS and RNS-based radicals. Furthermore, curcumin is a potent metal-chelator and inhibits the formation of NFT.

The inhibition of A*β *formation and aggregation is arguably amongst the most rational strategies employed to treat AD since it is established that A*β *is the key component to trigger AD pathogenesis. Considerable attention have been focused on the AD therapeutic potential of several plant derived polyphenolic compounds such as curcumin, tea extract and *gingko biloba*. Curcumin, a recognized pleiotropic natural compound is earmarked with strong potential to treat AD initially based on the epidemiological evidence in Indian population whereby long-term consumption of curcumin showed a 4.4 fold reduced incidence rate of AD compared to the USA [35]. Since then, compelling evidences from *in vitro*, *in vivo *and pre-clinical studies have systemically removed traces of lingering doubts regarding the therapeutic effect of curcumin in AD [17, 39, 40, 245-249]. Out of 214 anti-oxidant compounds tested in preventing fibrils formation, curcumin demonstrated the strongest inhibitory effect (IC_50_ = 0.25 μg/mL) [36]. The binding affinity of curcumin on A*β* aggregates was at least on par with the molecular imaging probes such as PiB in FDG PET with a *K_i_* of 0.07 nm for F18 labeled curcumin 0.679 μM) [37]. Curcumin could be able to bind to other β-pleated sheet structures including prion aggregates, synuclein and tau aggregates [38]. Curcumin showed dose-dependent inhibitory effect on the formation of A*β* fibrils with an EC_50 _of 0.09–0.63 μM [39]. In addition, the destabilization of preformed A*β* fibrils by curcumin was also dose-dependent at 0.1–1.0 μM [39]. Curcumin, demethoxycurcumin, bisdemethoxycurcumin displayed superior protective effect against A*β*_1–42 _insults with ED_50 _values of 3.0–7.1 μg/mL compared to α-tocopherol [250]. In another study, novel isolated curcumin compounds from natural products exhibited neuroprotective behaviour from A*β* (ED_50_ = 0.5–10 μg/mL) compared to Congo red (ED_50_ = 37–39 μg/mL) [251]. Similarly, the potent anti-amyloidogenic activities of turmeric extracts enriched with curcuminoids (IC_50 _= <5 μg/mL) are in accord with previous published data [247] (Fig. **6**). 

Using high level ab initio computational study, the key structural features of curcumin that facilitates the penetration into BBB and binding to A*β *were identified [252]. The analysis of bond charges revealed that the enolic and phenolic groups of curcumin are polar; and are essentially separated by a neutral hydrophobic conjugated carbon bridge, in which the latter facilitates the penetration into BBB. Meanwhile, the polar groups provided a site for deprotonation and subsequent binding site for A*β *oligomers [252]. 

Recent study by Zhang *et al*. demonstrated for the first time the reduction of A*β* levels *via *attenuation of APP maturation in the secretory pathway [253]. At least 40% reduction of A*β*_40 _and A*β*_42 _levels in mouse primary neuron cells were observed in conjunction with the administration of 20 μM curcumin. In addition, curcumin disrupted the APP maturation cycles by altering the turnover rate of immature APP to mature APP. In the presence of curcumin, the ratio of mature APP to immature APP was decreased by 67% possibly due to the increased stability of immature APP. Meanwhile, the effect of brefeldin A (BFA) that acted as Golgi-disrupting agent on APP maturation and trafficking was significantly attenuated by curcumin. In AD patients’ brain, immature APP exit endoplasmic reticulum (ER) and undergo *O*-glycosylation in Golgi complex to become mature APP. This mature APP is subsequently sorted onto plasma membrane after which it can undergo endocytosis *via *clathrin-coated pits. Consequently, the disruption of APP trafficking by BFA could significantly contribute to AD pathogenesis. Collectively, this study suggested the possible roles of curcumin to delay the exit of immature APP from ER, stabilizing immature APP at ER and simultaneously attenuated the endocytosis of APP from the cell surface [253].

Lin *et al*. independently showed that curcumin markedly attenuated the overexpression of APP and BACE1 in PC12 cells grown in the presence of metal ions such as Cu^2+^ and Mn^2+^ [254]. However unlike Cu^2+^ and Mn^2+^, Zn^2+^, both Fe^3+^ and Al^3+^ did not contribute to increased transcription levels of APP and BACE1. It is interesting to note that compared to other anti-oxidants such as minocyline and sodium ferulate, the authors found that curcumin demonstrated superior inhibitory effects on complete suppression of the transcription of both APP and BACE1. In addition, curcumin also possessed comparative ability to prevent up-regulated APP and BACE1 transcriptions with pathway inhibitors such as MEK-1, MAPK and JNK [254].

Different approaches have been devised to inhibit the degree of A*β* aggregation using curcumin, which include basic structural modification of curcumin that retained its anti-oxidant, anti-inflammation and anti-A*β* aggregation properties [246, 255-258] and formulation of nano-sized conjugated-curcumin [259, 260]. Briefly, the structure of curcumin consists of two aromatic end groups and a linker region in the middle (Fig. **1**). Reinke and Gestwicki previously described the structure-activity relationship of curcumin ligands with A*β* inhibitory activity [255]. The authors showed that simple aromatic group of curcumin did not have A*β* inhibitory activity while modification or loss of hydroxyl groups in the aromatic rings of curcumin abolished the effect, thus suggesting that aromatic substitutions capable of forming hydrogen interactions is crucial to maintain the binding activity of curcumin ligands to A*β*. In addition, the binding affinity of curcumin towards A*β *is influenced by the length and flexibility of linker region in which the optimal length lies between 8–16 Å [255]. With these knowledge in mind, Chen *et al*. designed novel curcumin analogues by varying the linker flexibility and substituting polar *N*-methylpiperazine on the two aromatic ends to allow the occurrence of hydrogen bonding and electrostatic interactions [246]. Curcumin analogue [3,5-bis-(4-(4-methylpiperazin-1-yl)benzylidene)-piperidin-4-one)] or labelled as compound A4 showed the most potent activity with approximately 90% of anti-A*β *aggregation activity and IC_50_ of 2.5 μM [246]. In addition, compound A4 also inhibited or delayed the A*β *conformational changes from α-helix to β-sheet structure and demonstrated significantly superior anti-oxidant activity compared to the unmodified curcumin and Trolox [246]. On the other hand, curcumin-derived pyrazoles and isoxazoles synthesized *via *the replacement of 1,3-dicarbonyl moiety of curcumin with these isosteric heterocycles showed 10 to 100 folds enhancement in its potency compared to unmodified curcumin. In addition to enhanced binding affinity towards A*β*_42_ aggregates and potent γ-secretase inhibitor, these curcumin analogues also served as depolymerizing agents for tau aggregates and could potentially inhibit the initiation of tau aggregation [256]. Besides that, a water-soluble curcumin conjugate, achieved by attaching a sugar moiety to curcumin, demonstrated amplified bioactivity by 1000 folds in comparison with unmodified curcumin [261]. Furthermore, this sugar-curcumin conjugate was effective against inhibition of tau peptide and A*β *aggregation at nano-molar concentration [261]. 

In another study, Orlando *et al*. chemically synthesized curcumin analogs with several modifications on the basic structure of the curcumin [257]. Several structural features had been identified contributing to anti-aggregation of A*β*. Their study concluded that a) at least one enone group in the spacer between aryl rings would be important for the anti A*β* aggregation, b) unsaturated carbon spacers between aryl rings would be needed for the same inhibitory effect, and c) substitution of methoxyl and hydroxyl at meta- and para- positions on the aryl rings showed improved anti A*β* aggregation effect. The authors also found that curcumin analogue 2 in which the para-hydroxyl group was substituted with methoxy (1,7-bis (3,4-dimethoxyphenyl)-1,6-heptadiene-3,5-dione) showed the highest inhibitory effect (6.7 folds higher) followed by curcumin analog 1 1,7-bis (3-hydroxy-4-methoxyphenyl)-1,6-heptadiene-3,5-dione (meta-and para-sub>stituted methoxyl and hydroxyl groups in reverse position as compared to curcumin) and curcumin analogue 8 [1,5-bis (3,4-dihydroxyphenyl)-1,4-pentadiene-3-one (contain hydroxyl groups in meta and para-positions of the aryl rings)] [257].

Curcumin-decorated liposomes with mean vesicle sizes of 131–207 nm were synthesized and evaluated for their integrity, stability and binding affinity to A*β*_1–42_ fibrils [259]. Both curcumin nanoliposomes synthesized using click chemistry method or conjugation with phospholipid displayed sufficient stability in plasma proteins and *in vivo *and the highest observed affinity to A*β*_1–42_ fibrils to date [259]. In another study, various curcumin associated nanoliposomes that were functionalized with lipid ligands (phosphatidic acid, cardiolipin, or GM1 ganglioside) showed promising results in binding to A*β* and subsequently interfering with the amyloid aggregation process through inhibition of oligomeric A*β* formation *in vitro* [262]. In a recent study, the targeting delivery of poly lactic-co-glycolic acid (PLGA) encapsulated curcumin nanoparticles (NPs) to central nervous systems was facilitated by the attachment of a Tet-1 peptide, a 12-amino acid peptide with binding characteristics to tetanus toxin, to the NPs. The Tet-1 conjugated curcumin NPs while retaining the potent anti-oxidant and anti-amyloid activities of curcumin had also achieved an enhanced uptake by neuronal cells [260]. In another study, amyloid-binding aptamers conjugated PLGA encapsulated curcumin NPs were employed to study the possibility of effective binding to amyloid plaques and thereby inhibiting its aggregation [263]. As demonstrated by the authors, the presence of aptamers ligand coupled with the anti-amyloid activity of curcumin showed effective targeting, binding to amyloid proteins and subsequently degradated amyloid into smaller fragments, which could be cleared by phagocytosis [263]. 

The anti-oxidant property of curcumin is believed to derive from its phenol moiety. As such, the donation of H-atom from *β*-diketone moiety to lipid alkyl or lipid peroxyl radical is considered the potentially more reliable mechanism underlying curcumin’s anti-oxidant activity. For instances, the donation of H-atom to a linoleic acid (bis-allylic radical) results in resonance stabilized β-oxo-alkyl curcumin radical with unpaired electron density distributed between three carbon and two oxygen atoms. The resonant curcumin radical undergoes molecular reorganization rapid intramolecular H-shift to generate the phenoxyl radical [264, 265].

Curcumin could scavenge NO-based radicals ultimately protected brains from oxidative damages (Fig. **6**). It had also been shown that curcumin prevented oxidative damage of DNA in mouse fibroblasts *via *scavenging of hydrogen radicals to prevent 8-hydroxy-2-deoxy-guanoise formation in the DNA molecule. Meanwhile, higher curcumin dosage induced mitochondrial and nuclear DNA damages to human hepatoma G2 cells whereby lipid peroxidation and ROS levels were markedly elevated under conditions of high curcumin doses [266]. Furthermore, curcumin protected rat 

cortical neurons from tert-butyl hydroperoxide induced oxidative damage through the reduction of ROS generation and DNA fragmentation [267].

Curcumin treatment significantly enhanced the levels of heme oxygenase-1 (HO-1) in brain cells [268, 269]. HO-1 is a ubiquitious and redox-sensitive inducible protein; responsible for the protection of various tissues from cellular-mediated stress and therefore its expression is an automatic response to increasing accumulation of toxic free heme levels in the brain. Together with its isozymes HO-2, HO-1 degrades heme *via *cleavage of heme’s ring at α-methene bridge to form biliverdin, carbon monoxide (CO) and free iron. subsequently, biliverdin is reduced to bilirubin by bilverdin reductase and serves as intracellular anti-oxidant agent. Both biliverdin and CO play important roles in modulating physiological process within nervous systems including inhibiting platelet aggregation and protecting brains tissues against oxidative injury [19]. HO-1 genes have consensus AP1, AP2 and NF-κB binding sites in its promoter region and are transcriptionally regulated by various stimuli including heme, oxidative stress (ROS/RNS), TNF-α and IL. As mentioned, curcumin transcriptionally activated HO-1 activity and HSP70 simultaneously and subsequently increased the tolerance of brain to stress [270]. The increase of HO-1 levels was not only due to the neurofibrillary tangles but also co-localized with senile plaques in AD brain. Thus once HO enzymes were activated by curcumin, it led to subsequent activation of ARE and Nrf2 pathways to neutralize both the oxidative and nitrosative stresses [271, 272]. 

Besides that, curcumin mitigated the glucose oxidase-mediated oxidative damage in rat astrocytes and cultured hippocampal neurons *via *induction of HO-1 activity in a dose and time-dependent manner [268]. As observed by the authors, significant elevation of quinone reductase and glutathione *S*-transferase levels were found in tandem with the increase of HO-1 activity and activation of Nrf2/ARE pathways. The expression of HO-1 reached maximal level after 6 h incubation with 15 μM of curcumin. The pre-treatment with 15 μM curcumin provided additional protection to cells as evident from the 70% recovery in cells viability that was exposed prior to oxidative damage. Meanwhile, at 100 μM, curcumin induced substantial cytotoxic effect to neuron cells as more than 4 fold reduction in cell viability was observed after 24 h treatment. Additionally, curcumin stimulated the activation of MAPK pathway which in turn induced the expression of heterodimers of NF-E2-related factors 2 (Nrf2) that is responsible for ARE activation reporter genes [268]. 

Curcumin protected PC12 against A*β*_25–35_-mediated toxicity in a dose dependent manner whereby pre-treatment with 5–10 μg/mL of curcumin restored cell viability. At concentrations above 50 μg/mL, it was observed that the cytoprotective effect of curcumin switched to cytotoxic instead [273]. Only low dosage of curcumin (9 μg/mL) was needed to scavenge 50% free radicals and to markedly reduce DNA damage in PC12 cells. Tau hyperphosphorylation induced by A*β *was also attenuated using curcumin treatment [251, 273] (Fig. **6**).

Previous data showed that curcumin reduced oxidative-stress related damage in rat forebrain [274]. The ischemic cerebral damage is related to excessive activation of *N*-methyl-D-aspartate. The treatment of rat retinal neurons with curcumin for 24 h reduced *N*-methyl-D-aspartate mediated oxidative damage and attenuated the intracellular accumulation of Ca^2+^ as observed from the increased cell viability and reduced apoptosis [275]. Park *et al*. reported similar observations whereby the intracellular concentrations of Ca^2+^ in A*β*-treated PC12 cells reduced after curcumin treatment [273]. 

The regulation of lipid peroxidation is crucial in the development of AD pathologies. Previous study demonstrated that curcumin protected human blood lymphocytes cells from negative side effects such as lipid peroxidation and DNA damage that occurred with the administration of mitomycin C (an antitumor agent) [276]. In addition, metal ions such as Al^3+^, Zn^2+^, Cu^2+^ and Fe^2+^ readily produced free radicals that translated into induction of lipid peroxidation, mitochondrial oxidation etc, thus underlining the potential therapeutic effect of metal chelators such as curcumin [277-284]. Zhao *et al.* demonstrated that curcumin could easily chelate Zn^2+^ and Cu^2+^ and the free radicals scavenging ability of resulting Cu^2+^-curcumin complex appeared to be stronger than curcumin alone [285] (Fig. **6**). Furthermore, the Fe^2+^ chelation activity of curcumin was studied at molecular levels in which mRNA levels of ferritin H and L and GSTα in liver cells increased in the presence of curcumin [286]. The activity of transferrin receptor 1 (protein stabilizer during iron limitation) and iron regulatory proteins (IRPs) were increased when incubated with curcumin [286]. García *et al*. evaluated the extent of DNA damage prevention (using *E. coli* DH1 purified plasmid DNA) with different metal ions chelator anti-oxidants such as catecholamine, amino acid, _L_-dihydroxyphenylalanine and curcumin. In their study, curcumin conferred significant protection to Fe^2+ ^andCu^2^-mediated damage with IC_50_ (µM) at o 6.2 ± 0.2 and 30.8 ± 0.6, respectively [287]. 

Recent study has also showed that curcumin was capable to protect mouse neuroblastoma neuro-2A cells from H_2_O_2_-induced oxidative stress and death (Fig. **6**). The pre-treatment of cells with curcumin increased cell viability to approximately 65% while reduction in apoptosis by almost 2-folds was recorded [288]. Curcumin treatment was also accompanied with increase in molecular expression of PARP and Bcl-2 in which the latter caused a decrease in mitochondrial permeability and slowed-down the release of cytochrome C [288]. In another study, the activation of NFκB and release of IL-8 in alveolar epithelial cells (A549) after induction with 100 µM H_2_O_2_ and 10ng/mL TNF-α were lower after treatment with curcumin [289]. In addition, glutathione and glutamylcyysteine ligase catalytic mRNA expressions were elevated with curcumin treatment in response to glutathione depletion [289]. The neuro-protective effects of curcumin against A*β*-induced neuronal oxidative stress, included scavenging free radicals, attenuation of oxidative damage from H_2_O_2_ and ROS and inhibition of* β*-sheeted aggregation [290]. 

As mentioned above, PS 1 is central for γ-secretase activity and also serves as substrate for glycogen synthase kinase-3β (GSK-3β). In normal condition, GSK-3β enzyme is involved in glycogen metabolism, cell cycle regulation and cell proliferation. However, over-expression of this enzyme is associated with AD development. Curcumin treatment in neuroblastoma cells significantly reduced both PS1 and GSK-3β expressions in a dose- and time-dependent manner. The authors showed that the inhibition of GSK-3β caused a reduction in PS-1 activity and subsequently inhibited the generation of Aβ_40_ and Aβ_42_ [245]. 

Curcumin is a recognized pleiotropic compound with ability to interact with various pro-inflammatory molecules secreted during inflammation process (Fig. **6**). Curcumin is neuroprotective towards lipopolysaccharide-stimulated activated microglial cells in a dose dependent manner with an IC_50 _of 3.7 μM. Curcumin suppressed the chronic and acute inflammation state by directly scavenging NO while the molecular targets of curcumin include cyclooygenase-2 (COX-2), TNF-α, IL-1, IL-2, IL-6, IL-8, IL-12, NFκB and MAPK activities [291]. In another study, pre-treatment with 10 µM curcumin showed positive effects in controlling microglia activity by deactivating NFκB and AP-1 pathways and inhibiting the gene expressions of pro-inflammatory factors such as TNFα, iNOS, COX-2, PGE_2_ and IL-1*β* [292]. In addition, curcumin is a potent modulator of microglial transcriptome as it effectively triggered anti-inflammatory signals while inhibiting the activation of BV-2 microglial cells migration [293]. Similarly, the authors reported that treatment of curcumin on lipopolysaccharide-stimulated microglial cells attenuated the transcription of NO synthase and corresponding signal transducers [293]. In comparison, demethoxycurcumin exhibited stronger inhibitory effect on NO and TNF-α production in lipopolysaccharide-stimulated primary microglial cells compared to curcumin and bisdemethoxycurcumin [294]. Recent evidence suggested that the neuroprotective effect of curcumin is mainly attributable to its anti-inflammatory properties especially *via *the inhibition of microglial cell activation [295]. The authors found that curcumin did not exhibit apparent protection towards dopamine-induced neuronal cell death but instead blocked the generation of pro-inflammatory and cytotoxic mediators [295].

### *In Vivo* Pharmacological Effect of Curcumin in AD Treatment

Using multi-photon microscopy approach, Garcia-Alloza et al. demonstrated the evidence of A*β* oligomers and fibrils suppression by curcumin* in vivo*. Curcumin administered (7.5 mg/kg/day) intravenously through tail vein for a week crossed BBB, retained in the vicinity of senile plaques and subsequently acted to simultaneously to reduce approximately 30% of plaques size and prevent the formation of new A*β*. In addition, curcumin conferred significant reversal of structural changes especially in the straightening of abnormal distorted neuritic curvature near the plaques. Curcumin treatment in APPswe/PS1dE9 mice also showed the tendency to decrease the level of soluble A*β*_40 _while concurrently soluble A*β*_42 _level was increased, which could be attributable to the insufficient time (duration of experiment was a week) to achieve significant biochemical changes of A*β* despite the observation of significant A*β *clearance [40]. As the treatment of curcumin in APPSw mice was prolonged to 6 months both soluble and insoluble A*β* were decreased. Lim *et al.* reported that low dosage of curcumin (160 ppm) administered orally for 6 months reduced both the insoluble amyloid and A*β* plaque burden in APPSw brain respectively by 39% and 43.6%, which were consistent with previous observation in ibuprofen-treated animals [41]. The authors also demonstrated significant suppression of oxidized protein and pro-inflammatory IL-1β levels after administration of both low and high doses of curcumin. Meanwhile, the well tolerance of AAPSw mice towards high curcumin doses indicated that this polyphenolic compound might be safer compared to (non-steroidal anti-inflammatory drug) NSAID since the complications usually associated with excessive use of ibuprofen such as gastrointestinal, renal and liver toxicities were apparently lower in curcumin treatment [41]. Furthermore, curcumin also acted as early intervention to limit the accumulation of amyloid *in vivo *as it bound directly to small *β-*amyloid species, which in turn successfully hindered the aggregation and formation of fibril [248]. Curcumin is a potent anti-amyloidogenic agent and is equally effective in clearing existing plaques in aged Tg2576 mice, which prior to treatment harboured massive amount of advanced state amyloids (greater than human brain AD) [248]. A daily single dose of curcumin (500 ppm) administered for 5 months to transgenic mice significantly reduced the level of insoluble A*β* and A*β* plaques. In old female Sprague-Dawley rats, curcumin treatment decreased the rate of A*β* deposition and despite receiving an intracerebroventricular infusion of A*β*, both pre- and post-synaptic protein levels increased by orally administrating 500 ppm curcumin for 2 months prior to A*β* infusion. It is interesting to note that very low dosage of curcumin was sufficient to reduce the level of insoluble and soluble A*β*, as well as plaque burden in mice cortex and hippocampus. However, for rats treated with high doses of curcumin, IL-1β and oxidised protein levels were reduced whereas no significant difference in A*β* deposition was observed thus indicating that the mechanism of action of curcumin had different dose requirements that could counterbalance each other at higher dosages [296]. 

In another study, the effectiveness of curcumin to alleviate A*β*-induced neurotoxicity was compared with ibuprofen in 22 month old Sprague-Dawley rat model [297]. In their study, infusion of lipoprotein carrier-mediated intracebroventricular was used to generate oxidative damage, synaptophysin loss and neuroinflammation and excess A*β *deposition. The dietary of 200 ppm curcumin suppressed oxidative stress and synaptophysin was maintained at normal level, while ibuprofen treatment did not yield the same result. The authors further demonstrated that a diet of 500 ppm curcumin thwarted A*β*-induced spatial memory deficits and A*β *deposition [297]. Recent evidence suggested the role of brain insulin receptors in the pathophysiology of dementia in Alzheimer’s disease. Based on this mechanism, researchers used streptozotcin to simulate the dementia symptoms in rat [298-300]. In a study using intracerebroventicular administered streptozotocin-induced dementia in rats, both pre- and post- treatment of curcumin (200 mg/kg) restored memory functions and brain insulin receptors [299]. In addition, curcumin treatment attenuated the streptozotocin-induced oxidative stress by reducing the acetylcholinesterase activity and enhancing GCH content in hippocampus and cerebral cortex [299]. These findings were supported by Awasthi *et al*., where the administration of curcumin (10, 20 and 50 mg/kg) to streptozotocin-induced memory impaired rats resulted in significant recovery of memory and attenuation of oxidative damage [300]. 

As it has been established, activated JNK is present in neurons and dystrophic neurites of both mice and brain AD models, which coincides with phosphorylation of protein insulin receptor substrate-1 (IRS-1) and tau, deposition of amyloid plaques and synapthophysin loss [301]. The treatment of 3xTg-AD mice with high-fat diet of DHA-rich fish oil or curcumin or a combination of both for 4 months suppressed the activities of JNK and phosphorylation of both IRS-1 and tau [302]. Administration of either curcumin or tetrahydrocurcumin to aged Tg2576 APPsw mice or lipopolysaccharide-injected wild-type mice reduced JNK activity and soluble A*β *accumulation [303]. Meanwhile, curcumin but not tetrahydrocurcumin had significant effect in the prevention of A*β *aggregation, which indirectly indicated that the presence of dienone bridge in curcumin was needed to reduce senile plaque deposition [303]. 

Recent studies demonstrated that curcumin increased lifespan and reduced A*β* neurotoxicity in invertebrate model systems [304, 305]. As demonstrated by Caesar *et al.,* using a transgenic* Drosophila* model, up to 75% improved lifespan and locomotor activity was observed for curcumin-fed flies. Meanwhile contrary to the majority published results regarding the effect of curcumin on A*β*, in this study no significant reduction in A*β* deposition was detected following curcumin administration [304]. The authors reported that curcumin accelerated conversion of pre-fibrilllar to A*β*_1–42 _in treated flies, which ultimately resulted in reduced neurotoxicity.

Orally administered curcumin increased the glutathione-*S*-transferases levels in mouse liver. The increase was slight (1.4 folds) albeit a high dose (500 mg/kg/day) of curcumin was adminstered for 14 days [306]. Contrary, in another study, Piper *et al*. demonstrated that low concentrations of curcumin (25-50 mg/kg/day) were sufficient to generate the 1.5 folds increase in glutathione-*S*-transferases levels in rodent livers [307]. The effect of curcumin in combating the effect of aluminium (Al^3+^) in promoting lipid peroxidation, PKC and AChE activities as well as lowering superoxide dismutase, glutathione peroxidase, glutathione-*S*-transferases and Na^+^, K^+^-ATPase activities were studied in different animal models. In a study conducted by Sharma *et al.*, curcumin treatment improved the activities of superoxide dismutase, glutathione peroxidase, glutathione-*S*-transferases in 10 and 24 month old Al-fed rats compared to control group in which the activities of the enzymes were significantly retarded by Al^3+ ^[308]. In another study, Kaul and Krishnakanth reported more than 100% increase in Na^+^ and K^+^-ATPase activities in brain microsomes in curcumin-fed rats [309]. Meanwhile, Bala *et al*. suggested that the ability of curcumin to promote Na^+^, K^+^-ATPase activities was beneficial for the excitability of the aged neuronal tissue [310]. Ataie *et al*. showed that intracerebroventricular administration of homocysteine (0.2 mmol) in rat brain increased malondialdenyde and superoxide anion levels, which subsequently led to lipid peroxidation, memory deficits and eventually cell death [311]. In their study, curcumin was used as a neuroprotective agent against oxidative stress and significantly improved the learning and memory deficits [311]. 

Curcumin alleviated the Al^3+^-induced oxidative damage and prevented mitochondrial dysfunction in rat brain [312]. As reported by the authors, administration of 50 mg/kg/alternate day of curcumin in conjuction with Al^3+^ (100 mg/kg/day) for a period of 8 weeks significantly normalized the altered activities of mitochondrial complexes and reduced the levels of glutathione in rat brain. For instance, the activities of NADPH dehydrogenase (complex I), succinic dehydrogenase (complex II) and cytochrome oxidize (complex IV) were lowered in three different regions of rat brain (cerebral cortex, mid brain and cerebellum). Curcumin is also reported to confer modulatory effects in Na^+^and K^+^-ATPase activities in brain microsomes. Researchers have found that phospholemman (PLM), a protein associated with Na^+^ and K^+^-ATPase, was abundant in selected area of central nervous system, thus suggesting the role of PLM to activate Na^+^and K^+^-ATPase activity [313]. Both Na^+^ and K^+^-ATPase are sensitive lipid peroxidation and since curcumin possessed antilipidperoxidative activity, it is believed that curcumin takes part in the activation process of Na^+^ and K^+^-ATPase. Owing to that, curcumin acted to counteract the Al^3+^-induced reduction of Na^+^ and K^+^-ATPase and to increase levels of protein kinase C (PKC) activities in adult and old rat brains [308]. The authors showed that co-administration of curcumin with Al^3+^ repressed the activities of phosphorylase kinase, protamine, PKC and AChE, while superoxide dismutase, glutathione peroxidase, glutathione-*S*-transferases and Na^+^, K^+^-ATPase levels were significantly elevated [308]. 

The role of curcumin as anti-oxidant and iron binding agent was compared with capsaicin and *S*-allylcysteine in Wistar rats [314]. Among them, curcumin had the highest oxidation potential (OP) (0.41V), followed by capsaicin (0.37V), ascorbic acid (0.012V) while *S*-allylcysteine did not show any anti-oxidant activity. However, when area under the curve (AUC) (proposed as a better parameter to measure total anti-oxidant) was taken into consideration, the activity of these compounds followed this decreasing trend: capsaicin (2.27 *µ*C) > curcumin (0.19 *µ*C) > ascorbic acid (0.45 *µ*C) > *S*-allylcysteine (0 *µ*C). Although ascorbic acid had lower AUC and OP values than capsaicin, ascorbic was more effective in scavenging DPPH radicals compared to capsaicin. Compared to capsaicin and *S*-allylcysteine, curcumin had the highest redox potential in reducing Fe^2+^ and QA-induced lipid peroxidation, which indicated that curcumin had stronger metal-ligand interaction and thus provided additional protection for redox cycling of iron and subsequently reduced lipid peroxidation. In terms of superoxide anion scavenging, the activity of curcumin is more significant compared to capsaicin and *S*-allylcysteine in scavenging the superoxide in rat brain homogenate regardless the used concentrations [314, 315]. The same authors also investigated the effects of curcuminoids (curcumin, demethoxycurcumin and bisdemethoxycurcumin) against Pb^2+^-induced neurotoxicity in male Wistar rats [315]. Rats co-incubated with Pb^2+ ^and curcumin/demethoxycurcumin for 5 days had higher glutathione content and less oxidised proteins compared to the control groups. In addition, curcuminiods-treated rats retained the spatial reference memory as seen from their faster escape latencies [315]. 

Central administration of colchicines caused increased in ROS generation, cognitive dysfunction, microtubule dysfunction and loss of cholinergic neurons in brain [316, 317]. In a recent study, the effect of curcumin against colchicines-induced cognitive and oxidative stress was determined in Wistars rats and compared with rivastigmine treatment. In the study, colchicine (15 mg/mL) was administered intracerebroventricularly to induce cognitive dysfunction. Pre-treated rats with curcumin (100, 200 and 400 mg/kg twice a day) for a period of 28 days before induction with colchicines showed improved protection against colchicines-induced cognitive impairment by improving the glutathione levels and AChE activities as well as reducing lipid peroxidation in colchicine-treated rats. Surprisingly, the ability of curcumin to control colchicines-induced cognitive impairment specifically in terms of protecting against oxidative stress seemed more potent than rivastigmine [318].

## PARKINSON’S DISEASE (PD) 

**Fig. (7):**
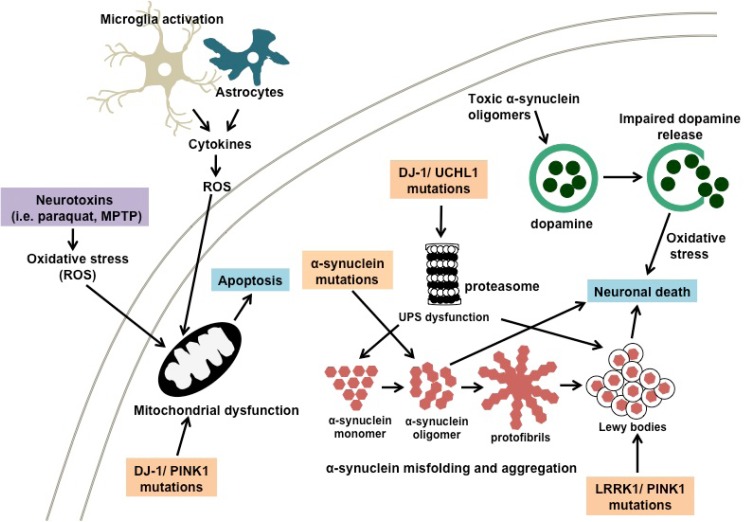
The neuropathological hallmarks of PD include the loss of DA producing neurons in the SN followed by the intracellular Lewy bodies accumulation in neurons. Lewy bodies are aggregates of normal, misfolded proteins which mainly contain abnormal 􀀁-synuclein. Exposure to neurotoxins such as paraquat, 6-OHDA, MPTP and rotenone result in generation of ROS and mitochondrial oxidative stress which subsequently cause apoptosis and possibly aggregation of 􀀁-synuclein. This also leads to several cellular and inflammatory responses such as the activation of microglia and astrocytes and release of pro-inflammatory cytokines. Mutations in DJ-1 and PINK-1 are known to cause mitochondrial dysfunction within the cells. Genetic mutations of α-synuclein, parkin and DJ-1/UCHL-1 lead to impaired UPS and higher accumulation of α-synuclein oligomers and Lewy bodies. Furthermore, toxic α-synuclein oligomers interact with DA vesicles and cause a surge in DA leakage which eventually leads to neuronal death.

PD, originally described by James Parkinson in 1817, is generally accepted as the second most common neurodegenerative disease after AD and affects approximately six million people worldwide. PD is a mysterious chronic disorder where its etiology still remains largely unknown despite the extensive effort to unravel the mystery. The typical clinical motor symptoms of PD include cardinal signs of tremor at rest, bradykinesia and rigidity instability [319, 320]. Meanwhile, signs of postural instability, gait dysfunction, speech difficulties and cognitive impairment manifest in the late stage of the disease [319, 320]. PD patients are also associated with non-motor symptoms such as depression, dementia, sleep difficulties, autonomic failures and pain, in which to date the exact mechanism of pain and its modulation is unclear. The neuropathological hallmarks of PD include the loss of dopamine (DA) producing neurons in the substantia niagra (SN) followed by the intracellular Lewy bodies and Lewy neurites accumulation in CNS (Fig. **7**). Lewy bodies and neurites, made up of aggregates of normal, misfolded proteins, mainly contain abnormal α-synuclein, which is prone to the formation of fibrils and aggregates [321-323] (Fig. **7**).

Majority of PD are sporadic, accounting to 90% of the cases, but familial PD is also observed in some cases. It is noteworthy to mention that the discovery of genes responsible for monogenic familial forms of PD has provided significant clues to the possible molecular and cellular mechanisms of PD pathogenesis. To date, eleven gene loci labeled PARK1–11, DJ-1, Parkin, leucine-rich repeat kinase 2 (LRRK2), ubiquitin carboxyterminal hydrolase-L1 (Uch-L1), α-synuclein and PINK1 have been characterized according to their function and cellular localization (reviewed in [324, 325]). Mutations of these genes consolidated the dysfunction of mitochondria (PINK1, DJ-1, Parkin and LRRK2) and ubiquitin proteasome system (UPS) malfunctions (Uch-L1, Parkin and α-synuclein) as key factors in PD pathogenesis (Fig. **7**). Although to date no model has been able to clearly summarize all the pathological trait of PD, the neurotoxin animal models of PD have achieved significant milestone contributing to our understanding of human PD. Neurotoxin models employed three neurotoxins such as 6-hydroxydopamine (6-OHDA), 1-methyl-4-phenyl-1,2,3,6-tetrahydropyridine (MPTP) and rotenone which best mimic the Parkinsonism *in vitro *and *in vivo*. 6-OHDA is recycled by dopamine transport and generates free radicals while MPTP is converted to 1-methyl-4-phenylpyridinium (MPP^+^) and accumulates in mitochondrial, thus leading to complex I inhibition. Rotenone is a complex I inhibitor. Extensive studies on these genetic and neurotoxic models have defined several molecular and cellular mechanisms such oxidative stress, α-synuclein aggregation, mitochondrial dysfunctions, neuro-inflammation and metal ions mediated toxicity as crucial events in the pathogenesis of sporadic form of PD. 

## MOLECULAR AND CELLULAR MECHANISMS INVOLVED IN PD PATHOGENESIS

### Altered DA Homeostasis and Oxidation

DA is particularly susceptible to oxidation to produce endogenous toxins, thus imparting oxidative stress on dopaminergic neurons. The homeostasis of DA is disrupted to a certain extent by α-synuclein oligomers which interacts with vesicles and results in a surge of DA leakage [326]. The altered DA homeostasis generates ROS and implicates mitochondrial functions; initiating subsequent events relating to oxidative stress (see section oxidative stress in AD). In particular, the accumulation of toxins MPP^+^ in DA neurons as a substrate for DAT damages dopaminergic cells, blockes mitochondrial respiratory chain, and subsequent activates cell apoptosis [327]. 

### α-synuclein Post Translational Modification, Aggregation and Fibril Formation

α-synuclein is a 140 amino acids protein, lacking both cysteine and tryptophan residues, which is abundant at presynaptic terminals as well as soluble regions and membrane-associated compartment of brain [42]. Under normal conditions, the possible roles of α-synuclein include synaptic vehicle release, fatty acid binding and neuron survival [328]. The focus of PD pathology is firmly entrenched in the aggregation of α-synuclein based on the following evidences accumulated over the years: a) autosomal dominant early-onset of PD occurred as a result of missense mutations in the α-synuclein gene or over-expression of wild type α-synuclein [329-331], b) the accumulation of α-synuclein in transgenic mice exhibited PD-like symptoms including motor impairment and inclusions [332], c) the deposits of α-synuclein were shown to be systemically detected in Lewy bodies and in animal models that were exposed to various neurotoxic compounds. α-synuclein is a flexible disordered protein, which could assume several structural conformations and aggregation states, either remaining unfolded or forming α-helix or β-sheets, depending on the environment and interactions with co-factors [43, 333, 334]. α-synuclein underwent rapid self-aggregation *in vivo *with an accelerated rate in the presence of transition metal ions, DA, proteins and lipids (Fig. **7**). The factors identified to promote α-synuclein aggregation *in vitro* were subtle changes in the environment (i.e. increase in temperature, decrease in pH), addition of amphipathic molecules such as herbicides, presence of external metal ions (industrial pollutants) and the interactions with membranes and other proteins [334-339] (Fig. **7**). For instance, studies showed that MPP^+^ and paraquat induced α-synuclein aggregation [338]. These factors may independently or synergistically stabilize a particular α-synuclein conformation and in turn induces different aggregation states such as soluble oligomers and insoluble fibrils (Fig. **7**). The dysregulation of α-synuclein concentration, existence of various aggregated states and post-translational modifications are believed to be the central of PD pathogenecity. Similar to AD, the soluble oligomers of α-synuclein seem the most neurotoxic. 

Spontaneous post-translational modification on α-synuclein, including phosphorylation, nitration, methionine oxidation, ubiquitination, AGE adducts formation, and acetylation had significant effect in the modulation of both the functions and aggregation of this protein (extensively reviewed [42, 43]). Smith *et al*. reported that the phosphorylation at Ser-129 residues of α-synuclein resulted in significant aggregation of α-synuclein [340]. In another study, *in vivo* reduction of Ser-129 phosphorylation with the introduction of phosphatase activity in transgenic mice, resulted in reduced α-synuclein aggregates that is in good agreement with the published data of Smith *et al*. [341]. It is interesting to note that not all phosphorylation induces α-synuclein aggregation. The phosphorylation site of α-synuclein at Ser-87 played a reversal role as it improves the protein’s flexibility and inhibites aggregation *in vitro *[342]. 

### Mitochondrial Abnormalities and Oxidative Stress

Abnormalities in mitochondrial function especially in the electron transport enzyme complex I deficiency is established in the SN of sporadic PD (Fig. **7**). subunits of enzyme complex I mitochondrial in PD brains suffered oxidative damage, malfunctions and were misassembled [133]. In addition, abnormal oxygen uptake and reduced complex I activity were also observed [343]. Neurotoxin models showed selective inhibition of complex I activity using MPP^+^ and rotenone that compromised the functions of mitochondria and generated ROS. Furthermore, covalent modification on various mitochondrial proteins by DA and its oxidised form DA-quinone (DAQ) heightened the mitochondrial swelling and proton permeability, ultimately resulting in mitochondrial respiration uncoupling and loss of ATP producing function [344]. Although the exact mechanism of DAQ-induced mitochondrial damage is unknown, it appeared that DAQ preferentially targets specific protein degradation through the binding to thiol groups of proteins. Proteomic analysis revealed that while majority of mitochondrial protein were unaffected upon exposure to DA, rapid loss of subset mitochondrial proteins including NADH dehydrogenase (75 kDa) and NADH oxidoreductase (30 kDa) subset of complex I, mitochondrial kinase, mortalin/GRP75, mitofilin, HSP60, peroxiredoxin 3 and voltage-dependent anion 2 were observed, many of which play critical role in tight regulation of mitochondrial homeostasis [345]. For instance, mitofilin served to maintain the integrity of mitochondrial while chaperone proteins HSP60 regulated protein folding and Fe-S protein assembly [346]. Meanwhile, both PINK1 and parkin acted synergistically for mitochondrial mitophagy function in which the former was responsible for localizing parkin into mitochondria [347]. Although mutations in both PINK1 and parkin did not exhibit major histopathological disabilites, mutated PINK1 transgenic mice were more susceptible to oxidative stress and ROS generations while parkin mutant genes sensitized DA neurons to cellular insults [348, 349]. DJ-1, localized in mitochondria, is a redox-sensitive molecular chaperone, which modulated oxidative stress responses. Similar to PINK1 and parkin, mutation in DJ-1 was not a causative agent for major PD abnormalities but its presence confered protection to counteract oxidative insult [350]. 

Oxidative stress in PD is associated with oxidative damages to proteins, lipids, mitochondria and decreased level in glutathione. Glutathione is synthesized in a two-step reaction involving γ-glutamyl cystein ligase (γ-GCL) and glutathione synthetase [351]. Glutathione is a tripeptide non-protein anti-oxidant and redox modulator in brain in which a drop in glutathione levels is an early indicator signaling oxidative stress in presymptomatic PD. Glutathione depletion observed in PD patients probably occurs *via *oxidation from increased ROS with significant damage to mitochondria. Jha *et al.* observed that glutathione depletion in dopaminergic cells increased oxidative stress and mitochondrial malfunction [352]. In addition to ROS, peroxynitrite (PN), a short-lived RNS produced in mitochondria, rapidly damaged proteins by modification of tyrosines into 3-NT and subsequently inhibition of respiratory complex activity. In a study by Murray *et al*., the authors reported dose-dependent inhibition of complexes I, II and V by PN treatment, where approximately 50% and total loss of activities were reported respectively using 800 and 2400 μM of PN, whereas complex III remained active or unaffected [353]. 

### Ubiquitin–proteasome (UPS) Dysfunction

UPS, a principal cellular proteolysis mechanism consisting of ubiquitin, is involved in the degradation of key regulatory non-lysosomal, abnormal, misfolded or unassembled proteins [354]. Therefore, slight malfunctions arising from overloaded or impaired UPS could lead to over-accumulation of toxic proteins, and eventually cellular death. The UPS dysfunction has been investigated *via *several cellular models and it appeared that increased oxidative stress or mitochondrial complex I inhibition are amongst the reasons behind the collapse of UPS pathway. The link between UPS impairment and PD pathogenesis is increasingly clear because in addition to α-synuclein, Lewy bodies also contain ubiquitin, parkin and other proteasomal proteins [355-357] (Fig. **7**). This is further consolidated with studies that showed the formation of α-synuclein inclusions reminiscent to PD pathogenesis in primary neurons of rat following the treatment with lactacystin, a protesomal inhibitor [358, 359]. In another *in vivo *study, systemic administration of proteasomal inhibitors to Sprague-Dawley rats also produced typical PD-like symptoms and pathological phenotype [360]. There is no doubt that the presence of proteasome in Lewy bodies is in somehow linked to the activation of UPS compensatory response by the α-synuclein aggregates during PD pathogenesis development. Direct evidence can be found by the observation of UPS malfunctions in the brains of sporadic PD patients. In a study by Tanaka *et al*., over-expression of both wild type and mutant α-synuclein inhibited the activity of UPS in dopaminergic PC12 cells [361]. In contrast, Martin *et al*. observed negligible effect on UPS function in mice and transfected PC12 cells with wild type and mutant α-synuclein [362]. 

The degradation activity of ATP-dependent UPS is severely affected owing to the impaired ATP generating ability of mitochondria upon continuous exposure to neurotoxins. Furthermore, the increased sustained intracellular ROS generations and oxidative insults by neurotoxins such as MPP^+ ^and rotenone, significantly augmented protein oxidation and could possibly oxidised proteasome [363]. In such events, the protein degradation capacity of proteasome could be greatly overwhelmed. In other words, these oxidised proteins were not degraded by proteasome rapid enough to avoid apoptotic death resulting from the build up of these proteins. The gradual decline of UPS functions could also be contributed to aging as observed in impaired UPS degradation in both aged human and animal tissues in CNS [364-366]. 

## CURCUMIN AND PD

**Fig. (8):**
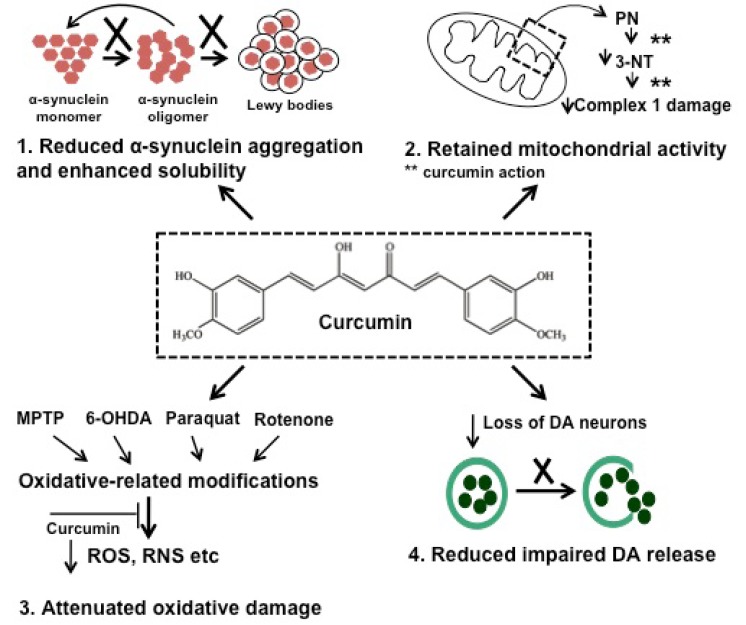
The neuroprotective role of curcumin against PD.

The neuroprotective role of curcumin in PD is increasingly clear as seen in the published result of Pandey *et al*., [50] which demonstrated the direct influence of curcumin on α-synuclein aggregation. In the presence of 1 mM Fe^3+^, curcumin conferred a dose-dependent inhibition on α-synuclein aggregation and simultaneously enhanced α-synuclein solubility [50]. It is also demonstrated that curcumin have the ability to disperse/break preformed α-synuclein aggregates (Fig. **8**). The authors further confirmed their *in vitro *findings with neuroblastoma cells and dopaminergic MN9D cells. However, it is unfortunate to note that the reduced α-synuclein aggregates were compensated with an increase of SDS-resistant oligomeric species, which could provoke counter-productivity [50]. In a recent study, curcumin alleviated the α-synuclein-induced cytoxicity in SH-SY5Y neuroblastoma cells by reducing the intracellular overexpression of α-synuclein and ROS generation [367]. Furthermore, the activation of caspase-3 was effectively inhibited and the signs of apoptosis were lessened [367]. In another independent study, using a different *in vitro* cell model, the effect of curcumin on α-synuclein also resulted in similar outcome. The addition of curcumin on an A53Y α-synuclein mutant PC12 inducible cell model of familial Parkinsonism attenuated the induction of cell death *via *reduction of intracellular ROS, mitochondrial dysfunction, cytochrome *c *release, caspase 3 and caspase 9 activation [368] (Fig. **8**). Meanwhile, a novel chemically synthesized curcumin-glucoside analogue prevented both oligomer and fibril formation and yielded superior binding affinity towards α-synuclein oligomers in a dose dependent manner [369]. Furthermore, the same compound solubilized the oligomeric form of α-synuclein by disintegrating the preformed fibrils based on the observation of enhanced α-synuclein solubility upon curcumin-glucoside addition [369].

Several *in vivo *and *in vitro* studies employed 6-OHDA and MPTP models to mimic the experimental disease of PD; because these models recapitulate the essential pathological and biochemical trademarks of PD including oxidative stress, mitochondrial dysfunction and DA degeneration. Administration of natural phenolic anti-oxidants including curcumin in a 6-OHDA rat model significantly attenuated the loss of DA neurons in SN [370]. Pre-treatment of curcumin in 6-OHDA rats resulted in reduced DA neuron loss (21%) compared to control group (about 50%). The authors also showed that post-treatment of curcumin to lesion attenuated the loss of DA metabolites, dihydroxypenylacetic acid and homovanilic acid in the striata [370]. The neuroprotective action of curcumin against 6-OHDA-mediated toxicity is associated with its anti-oxidant behavior [48, 370]. MES23.5 cells treated with curcumin showed partial restoration of mitochondrial activities, remarkably enhanced expression of Cu-Zn superoxide dismutase and reduced intracellular ROS accumulation [48] (Fig. **8**). In addition, the anti-oxidant action of curcumin was accompanied by the simultaneous inhibition of NFκB cytosol translocation to nucleus and subsequent inactivation of cell induced apoptosis genes such as p53 [48]. The anti-apoptotic feature of curcumin was further elucidated in a recent study by Jaisin *et al*., [371] which described the attenuation of p53 mediated apoptosis in SH-SY5Y cell lines using a 6-OHDA model. The prevention of 6-OHDA-induced DA loss and ROS generation by curcumin pre-treatment was dose-dependent, whereby cell viabilities in 20 μM curcumin and control group (without curcumin) was 92% and 59%, respectively. The protection conferred by curcumin against 6-OHDA toxicity was due to the inhibition of p53 phosphorylation and the restoration of balance between anti- and pro- apoptotic proteins of Bcl-2 family [371]. 

The findings of Chen *et al*. [49] clearly indicated that the molecular mechanism involved in the protection of curcumin against MPP^+^-induced apoptosis in PC12 cells is *via *Bcl-2-mitochondria-ROS-iNOS pathway. The survival rate of cells administered with 0.25 mmol/L curcumin and MPTP was 72% compared to only 45% cell viability in MPTP alone. The authors also found that the inhibition of ROS level and iNOS expression by curcumin is in tandem with the over expression and antagonization of anti-apoptotic protein, Bcl-2, thus suggesting a synergistic role of Bcl-2 in curcumin-induced protection [49]. Meanwhile, Rajeswari and Sabesan showed that DA depletion and enhanced monoamine oxidase-B (MAO) activity as a function of MPTP induced toxicity was attenuated with curcumin and tetrahydrocurcumin [372]. 

Studies have demonstrated that the activation of JNK signaling pathway induced by MPTP/MPP^+^ mediated-neurotoxicity and 6-OHDA lesion models in PD is involved in DA degeneration [373, 374]. Yu *et al*. found that curcumin inhibited JNK-mediated DA apoptosis induced by MPTP/MPP^+^ exposure in both C57BL/6N mice and SH-SY5Y cell models [375]. Curcumin alleviated the deficits behavior in mice as the locomotion and rearing frequencies of animals were enhanced. Additionally, neuron survival in mice was markedly improved while the activation of astrocytes was reduced using curcumin treatment. Both *in vitro *and *in vivo *models exhibited similar inhibitory effects of curcumin in the activation of JNK, c-Jun and caspase-3. Based on their findings, the authors suggested that in addition to anti-oxidant and anti-inflammatory properties, the intervention of curcumin in PD also involved complex regulatory of cell-induced apoptosis such as JNK pathway [375].

Dose-dependent neuropreventive activity of curcumin prior to PN-induced oxidative insult in spiral ganglion neurons had been observed in a study by Liu *et al*. [376]. Similar to previous findings, curcumin reduced apoptotic rate and expression of malonaldehyde while restoring the cellular levels of glutathione and superoxide dismutase. It should be noted that in their study, the upper limit of curcumin concentration was 15 μM as curcumin switched from anti- to pro-oxidant behavior, where enhanced PN-induced damage was observed beyond this concentration. 

Curcumin is also effective against the complex I damage mediated by PN accumulation in mitochondria [45]. The pre-treatment of curcumin protected brain mitochondria against RNS exposure *in vitro *by direct detoxification and inhibition of 3-NT formation. It is suggested that curcumin protected complex I *in vivo *indirectly through the induction of glutamate-cysteine ligase expression and glutathione synthesis. The elevation in glutathione levels in concurrent administration of curcumin in N27 dopaminergic neurons served additional protection against RNS stress. The same group further elucidated the mechanism of curcumin action using both experimental (*in vivo*, *in vitro*) and dynamic biological simulation mitochondrial dysfunction models. Both *in vivo *and *in vitro *experimental data demonstrated that curcumin protected GSH against depletion and mitochondrial damage-mediated oxidative stress in neuron cells while simulations showed that curcumin induced glutathione synthesis *via *increased γ-GCL transcriptions [45, 46]. Thus, it is envisaged that the restoration of glutathione could be a preventive step to avoid the trigger of early pre-symptomatic PD. Bioconjugates of curcumin displayed improved neuroprotection activity (in terms of compensating the glutathione level) compared to curcumin [377]. For instance, the restoration of glutathione levels after pre- and post- treatment of glutamic acid derivative of curcumin were 83% and 136%, respectively compared to curcumin alone (78% and 127%). In addition, curcumin bioconjugates also demonstrated higher bioavailability and ROS scavenging capacity in PD [18, 377]. The same group took a step further to demonstrate that curcumin bioconjugates offered superior protection against PNS-mediated RNS stress and damage to brain mitochondria [18].

Although the anti-inflammatory properties of curcumin are well established, not many studies relate their effect in molecular basis on neuronal systems. Jung *et al*. [291] investigated the underlying effects of curcumin treatment on NO generations and related signaling pathways in primary microglial cells activated by inflammatory stimuli such as LPS [291]. Their findings could be summarized into: a) generation of NO was regulated through inhibition of JNK, p38 and NFκB signaling pathway, and b) compared to other macrophages and cell lines in brain, the concentration and inhibitory effect of curcumin in primary microglia cells is predominant, thus implying the potential therapeutic application of this compound in neurodegeneration diseases associated with inflammation. In addition to that, other studies also independently reported similar findings albeit employing different *in vitro *and *in vivo *models [292, 378, 379]. For instance, curcumin inhibited the generation of glutamate, OH^-^, prostaglandins and activation of pro-inflammatory cytokines in rat’s hypothalamus [379]. Meanwhile, coincidentally the effect of curcumin treatment prior to LPS induced DA toxicity in primary rat neuron/glia cells was dose dependent as its administration lessened the generation of pro-inflammatory genes as well [292]. 

Although most studies had described the beneficial effects of curcumin in PD, a few contradictory reports had shown that curcumin actually exacerbated the disease, notably in a study by Ortiz-Ortiz *et al*., [380] which showed higher apoptosis rate in N27 mesencephalic cells after co-incubation in curcumin and paraquat. Curcumin seemed to sensitize the cells to paraquat-induced apoptosis and instead acted as pro-oxidant to greatly enhance irreversible ROS production. In another study, the exposure of N27 mesencephalic cells to curcumin induced the over-expression of LKKR2 in a time dependent manner, which thus implicating curcumin in the PD pathogenesis [381].

## BRAIN TUMOR

Malignant brain tumors are the second leading cause of death from disorders of neuron origin in the United States. Although the incidence rate of primary malignant brain tumors is relatively low compared to other tumors (approximately 6.6 per 100000) [382], nevertheless they are touted as the most lethal type of tumors with their general median survival period of less than 2 years. Furthermore, the mysteries surrounding the etiology of malignant brain tumors and relatively insufficient knowledge on the risk factors make its treatment extremely difficult. Malignant gliomas are the most common primary CNS tumors in the brain, which could be classified histologically as astrocytomas, oligodendrogliomas and oligoastrocytomas. Both oligodendrogliomas and oligoastrocytomas are sub-divided into grade II and III tumors while astrocytomas are classified into pilocytic astrocytoma (grade I), astrocytomas (grade II), anaplastic astrocytoma (grade III) and glioblastoma multiforme, GBM (grade IV). Low grade tumors such as pilocytic astrocytoma are slow growing; non-infiltrative tumors with median survival time of several years and are usually curable by surgery. Astrocytomas affected-brain tissues often display tendency to infiltrate into surrounding normal brain tissues, thus the complete successful removal of affected tissues/regions with surgery is hardly possible. GBM is the most aggressive, infiltrative and prevalent type of gliomas and is responsible for 30% of primary brain tumors and the highest death incidence among other primary brain tumors [383]. Patients with GBM have median survival of less than 1 year while those affected with grade II astrocytomas could live for 10 to 15 years [384]. GBM is subdivided into either primary or secondary GBM. Primary GBM arises *de novo* without evidence of malignant precursor lesions whereas secondary GBM is believed to develop progressively from low-grade gliomas, which have undergone series of genetic mutations [385]. In spite of the obscurity behind the complexity of complete molecular pathways involved in tumorigenesis, emerging evidences from molecular biology incriminated the involvement of genetic abnormalities which affect two fundamental cell control cycles and deregulation of signal transduction factors.

### Loss of Cell Control Cycles

**Fig. (9):**
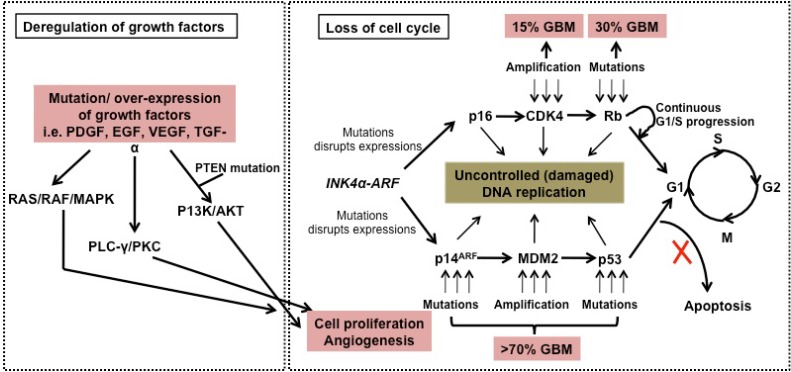
Primary GBM is associated with mutations of genes, which regulate RB/E2F pathway. In normal cell cycle control, the presence of DNA irregularities suppresses the p16-CDK4-RB pathway to inhibit progression of cell cycle past GI restriction point while p53 is activated to commit the damaged DNA to apoptosis. Contrarily, in diseased state, the activation of p53 is inhibited while uncontrolled replications of damaged/mutated DNA occur at the same time. In addition, the mutation or over-expression of growth factors and their corresponding factors leading to subsequent activation of downstream signaling pathways are also responsible to GBM.

In general, the duplication process of cells, commonly referred as cell cycles involves four phases: the S phase (the replication of DNA), M phase (mitosis), G1 (pre-DNA replication) and G2 (pre-mitosis). Two key regulatory pathways (p16-CDK4-RB and ARF-MDM2-p53 pathway) are involved in controlling cells duplication activities from unrestrained progression of cell cycles, whereby the principal proteins involved in these distinct pathways are retinoblastoma tumor suppressor protein (RB) and p53 tumor suppressor protein (Fig. **9**). In the p16-CDK4-RB pathway, RB is activated through phosphorylation by the cyclin dependent kinase (CDK) 4/6-cyclin D1 complex. However, phosphorylation of RB is blocked by cyclin dependent kinase inhibitors (CKI) such as p16^INK4A^ through inhibition of CDK 4/6-cyclin D1 complex activities [386]. These inhibitors are especially important in two ways: a) to ensure the DNA replication and cell division occurs at appropriate time and b) to stop the cell cycle progression in case of any irregularities such as mutated or damaged DNA. The phosphorylated and activated RB binds to transcriptor factors such as E2F families, leading to transcription of various genes involved in different cellular responses and particularly in the activation of cell cycle progression beyond the G1/S transition. 

p53 is activated in response to DNA damage and hypoxia, which thus induces either cell cycle arrest (inhibits cell cycle progression through G1 and G2) or apoptosis. p53 is regulated by murine double mutant 2 (MDM2) in which its binding to p53 inhibits the transcription and leads to rapid breakdown of p53. MDM2 in turn is inactivated by the binding to p14^ARF^, which ensures the elimination of damaged DNA from progressing through cell cycles. In other words, in normal cell cycle control, the presence of DNA irregularities suppresses the p16-CDK4-RB pathway (to inhibit progression of cell cycle past GI restriction point) while p53 is activated to commit the damaged DNA to apoptosis.

Primary GBM is associated with mutations of genes, which regulate RB/E2F pathway. For instance, mutations in both CDK4 (amplification) and RB1 are respectively observed in 15% and 30% of GBM [387-389]. Meanwhile, mutations in the *INK4a-ARF *locus disrupt the appropriate expressions of p16^INK4A^ and p14^ARF^. The inactivation of p53 pathway is frequent in GBM (more than 70%), which occurs as a result of p53 gene mutations, amplification of MDM2 and the loss of ARF [390] (Fig. **9**). 

### Deregulation of Signal Transduction Factors and their Corresponding Receptors

The mutated or over expressed growth factors and their corresponding factors and subsequent activation of downstream signaling pathways are often implicated in GBM. The platelet-derived growth factor (PDGF), epidermal growth factor (EGF), vascular endothelial growth factor (VEGF) and transforming growth factor-α (TGF-α) bind and activate their corresponding receptors with intrinsic protein kinase activity, whereby most often these receptors are receptor tyrosine kinase (reviewed in [391]). Upon binding to growth factors, these receptor tyrosine kinases are activated through dimerization and phosphorylation, which trigger the activation of three intracellular protein cascades: the RAS/RAF/MAPK (ERK) pathway, P13K/AKT pathway and PLC-γ/PKC pathway.

VEGF is considered the most important growth factor associated with glioma angiogenesis as it possesses highly specific mitogenic and chemotactic activity on endothelial cells. VEGF wields its activity through the binding to VEGFR. Phosphorylated VEGFR enhances endothelial cell protection and activation of protein kinase C and RAS/RAF/MAPK (ERK) pathway [392]. At least 50-fold over-regulation of VEGF was observed in glioma cells compared normal brain tissues [393]. This observation is in tandem with the hypothesis of paracrine tumorigenesis where the enhanced secretion of VEGF by tumor cells lead to rapid propagation of these affected cells. Several factors are established to affect the transduction signals of VEGF, which include P13K/AKT pathway during hypoxia conditions, RAS/RAF/MAPK (ERK) pathway during normoxia and probably *via *the activation of EGFR in non-hypoxia conditions. In addition, as VEGF promoter contains NFκB putative response element, its expression is therefore enhanced by NFκB. Indeed, it has been demonstrated that the inhibition of NFκB in glioma cell lines showed a decrease in VEGF expression, resulting in suppressed angiogenesis *in vivo *and *in vitro*. Other factors known to induce the expression of VEGF *in vitro *include phorbol esters, TGF-β, EGF and p53. PDGF (AA, AB and BB) are expressed by astrocytoma and their expression levels increase with increasing malignancies of tumor. For instance, PDGF-β was not expressed in normal brain tissue whereas it was induced in low-grade glioma and GBM in which the expression level was up regulated to a greater extent in the latter form. Both EGF and TGF-α bind to EGFR and are considered inducers for angiogenesis *in vivo*. Several studies have reported that signaling of EGF and TGF-α is prerequisite for sustained proliferation, survival and motility of neuron stem cells. The over-expression of constitutively active EGFR (known as EGFRvIII) is often present in GBM where it acts to activate P13K/AKT and RAS/RAF/MAPK (ERK) pathways. Furthermore, mutations of EGF genes (amplification) were observed in approximately 30–50% of primary GBM while the mutations of EGFR (located on chromosome 7p12) resulted in the binding failure of growth factor to mutant receptor [394]. The up-regulation of EGF coupled with loss of cell cycle control (both p16^INK4A^ and p14^ARF^) was shown to induce higher-grade glioma formation *in vivo*. 

## THERAPEUTICS IMPACT OF CURCUMIN ON BRAIN TUMORS

### Anti-proliferative Effects of Curcumin

**Fig. (10):**
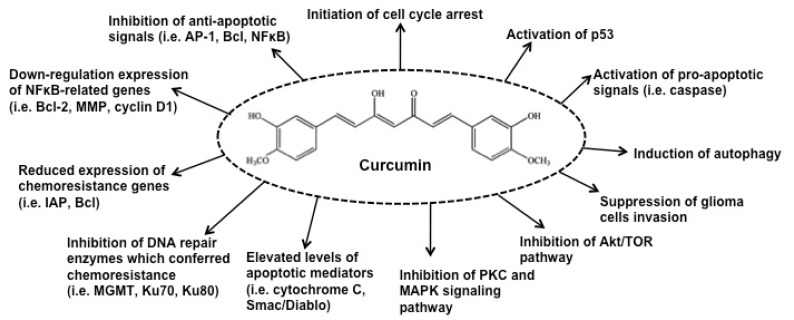
Curcumin as anti-tumor agent and its responding molecular targets.

Timiras *et al*. first reported the dose-dependent effect of curcumin in suppressing the proliferation of glioma cells *in vitro*. Since then, emerging evidence is slowly consolidating the anti-proliferative effects of curcumin on primary brain tumor progression. The anti-proliferative effects of curcumin usually occur through its effect on cell cycles in particular the initiation of cell cycle arrest and activation of p53. The activation of pro-apoptotic signals such as caspase family and inhibition of anti-apoptotic genes (AP-1, NFκB, Bcl) is among the typical responses observed in tumor cells upon curcumin treatment. Consequently, the down-regulated expression of NFκB-related genes including Bcl-2, MMP-9, cyclin D1, cyclooxygenase-2 and IL-6 initiates cell cycle arrest event, anti-proliferation and induction of apoptosis (Fig. **10**). 

Dhandapani *et al*. investigated the effect of curcumin on sensitizing and suppressing the growth of human glioblastoma cells [52]. Several interesting key findings of their study include: a) the cell death induction effect by curcumin was independently through p53 and caspase upregulation, which effectively dodge the atypical stimuli by both p53 and caspase that exert increased the survival and chemoprotection to tumor cells, b) the binding activities of AP-1 and NFκB were decreased (where the constitutively over-expression of these genes is a marker in GBM), c) curcumin decreased the expression of genes which conferred chemoresistance such as IAP and Bcl family, and d) the expression of DNA repair enzymes (MGMT, Ku70, Ku80 and DNA-PK_cs_) believed to be involved in chemosresistance and radioprotection was inhibited by curcumin. The authors also demonstrated that curcumin treatment prior to radio-therapy managed to sensitize the aggressive radioresistant GBM, thus opening new window of possibilities *via *synergistic activities between curcumin and chemotherapeutic agents to maximize cell death [52]. Besides up-regulating the expression of p53 in U251 glioma cells, the initiation of G2/M cell cycle arrest also induced ING4 expression, a novel member of ING tumor suppressor family when curcumin was employed [54] (Fig. **10**). 

In another study, curcumin reduced inherent resistance of human malignant GBM U87MG cells *via *inhibition of anti-apoptotic signals such as Bcl-2, IAPs and NFκB while simultaneously activated intrinsic and extrinsic caspase cascades, thus leading to tumor cell apoptosis [395, 396]. In addition, the authors found that the levels of cytochrome C and pro-apoptotic molecule Smac/Diablo released from mitochondria following curcumin treatment were increased. Both cytochrome C and Smac/Diablo are known mediators leading to apoptosis; where cytochrome C acts to activate caspase-9 while Smac/Diablo suppresses IAP expression in cytosol. The downregulation of Bcl-2 is implicated in curcumin-induced apoptosis and cell cycle arrest in G2/M phase. Luthra *et al*. took a step further to compare the effectiveness of curcumin and its curcuminoids derivatives (demethoxycurcumin and bisdemethoxycurcumin) in the treatment of human glioma U87 cells [397]. Similar to other published results, the authors showed that cascades of events leading to apoptosis, which involved anti-proliferation, chromatin condensation and DNA fragmentation was evident in curcumin, bisdemethoxycurcumin and demethoxycurcumin teatments, with the latter demonstrating superior activity compared to the other compounds. These compounds induced G2/M cell cycle arrest at micromolar concentration (12.5 μg/mL) after 24 h of administration. After 48 h, sub-G1 apoptotic fraction was evident for demethoxycurcumin while both curcumin and bisdemethoxycurcumin were still locked at G2/M arrest [397]. Dose-dependent apoptosis induced by demethoxycurcumin in human GBM 8401 cells is suggested to be linked to mitochondria- and caspase-dependent pathways [398]. The authors showed that demethoxycurcumin exerted cytotoxic effects on human GBM 8401 cells with IC_50_ of 22.71 μM. Similarly, the demethoxycurcumin treatment led to series of pro-apoptotic events, including induction of cell cycle sub-G1 arrest and DNA fragmentation, the activation of caspase and the loss of mitrochondrial membrane potential while the activity of NKκB remained dormant in human GBM 8401 cells [398]. 

Another type of programmed cell death is non-apoptotic autophagy, which is characterized by autophagic vacuoles in the cytoplasm. It was observed that although malignant glioma cells were very resistant to apoptosis, however they were susceptible to autophagy in response to anti cancer therapies. Aoki *et al*. demonstrated that curcumin induced G2/M cell cycle arrest and non-apoptotic autophagy in U87-MG and U373-MG cells [399]. The induction of autophagy was a result of Akt/mTOR/p7056K pathway inhibition and ERK pathway activation. In addition, curcumin also inhibited the growth of subcutaneous xenografts of tumor cells and induced autophagy. It is also interesting to note that the anti-tumor effect exerted by curcumin through autophagy was not caused by the inhibition of NFκB. Studies showed that poorly differentiated glioma-initiating cells were responsible for self-renewal and recurrence of GBM. Meanwhile, curcumin is shown to activate autophagy and induce differentiation cascade in glioma-initiating cells *in vitro *and *in vivo*, followed by decreased self-renewal and clonogenic ability [400] (Fig. **10**). 

### Suppression of Glioma Cells Invasion to Neighboring Normal Cells 

The infiltration of glioma cells to surrounding normal cells is mediated by the abnormal expression of MMP. Therefore, it is conceivable that the compounds which could inhibit or suppress MMP expression and corresponding upstream regulatory pathways would be used in the treatment of brain tumors. The suppression of MMP-9 expression in phorbol ester induced human astroglioma cells *via *inhibition of PKC and MAPK signaling pathways was exerted by curcumin [401]. The same group further revealed the broad-spectrum inhibitory behavior of curcumin, as the activities of other MMPs (-1, -3 and -14) molecules were simultaneously repressed in tandem with curcumin administration, which indirectly pointed out the reason glioma invasion was significantly restricted by curcumin. The suppressed expressions of these genes were a direct result of inhibition of the modulator, AP-1 transcriptional activities, by curcumin [402]. 

To date, studies on the *in vivo* efficacy of curcumin on primary brain tumors are selectively few. The *in vivo *inhibitory effects of curcumin had been established on other cancers such as skin, lymphomas/leukemia, colon and liver. The first report consolidating the anti-tumor role of curcumin *in vivo *was pioneered by Perry *et al*., [403]. The authors showed that the pre- and post- administration of curcumin exhibited significant anti-tumor effects with slower tumor growth and improved survival time of immune-compromised mice. In particular, the mechanism of its action *in vivo *was *via *the decrease of gelatinolytic activities of matrix metalloproteinase-9 (MMP-9). In addition, curcumin down-regulated CD31and CD105 mRNA (two endothelial cell marker from newly formed vessels) and reduced the content of hemoglobin in glioma cells. It was also noted that curcumin could penetrate easily through the tight junction of BBB, thus suggesting the partial reason contributing to its anti-tumor affect regardless of *in vitro* or *in vivo *model [403]. Meanwhile, Zanotto-Filho *et al*. reported their attempt to regulate the anti-glioma activity of curcumin using immune-competent rats [404]. Intraperitoneal administration of curcumin (50 mg/kg/day) decreased up to 80% brain tumors in rats with negligible damages observed in tissues (transaminases, creatinine, alkaline phosphatase). Furthermore, metabolic (cholesterol and glucose), oxidative and hematological toxicity was not detectable post-treatment of curcumin. In accordance with previous studies, curcumin affected *in vitro* the activation of P13K/AKT and NFκB pathways, inhibited the Bcl-2 expression and induced mitochondrial dysfunction in a p53 and PTEN independent manner [404]. 

## LIMITATIONS OF CURCUMIN

### Bioavailability of Curcumin in Animals and Human

The low bioavailability of curcumin is a major drawback, which hampers its development as an effective therapeutic agent. Over the years, extensive research had provided insights for poor curcumin bioavailability *in vivo, *including low absorption and serum levels, high metabolizing rate and rapid systemic clearance as confirmed in countless orally administered curcumin in human and animal models [405-407]. Relatively high number of reports had shown that plasma concentrations of curcumin administered *via *oral or intraperitoneal route were very low, typically in the nanomolar range (Table **4**). The first bioavailability, distribution and excretion of curcumin study using Spague-Dawley rats appeared in 1978 by Wahlstrom and Blennow [405]. It was concluded that the content of curcumin in rat’s blood plasma was negligible even though excess amount of curcumin (up to 1 g/kg) was administered orally. More than 75% of curcumin were excreted into the feces while traces amount was found in urine, which indirectly showed that the gut (caecum) of rat had poor curcumin absorption capability. In addition, no apparent side effects were observed on the rats even though high doses (5 g/kg) were administrated. In another separate study, Holder *et al.* attempted to increase the bioavailability of curcumin through intraperitoneal administration approach into mice [408]. However, this approach did not yield significantly improved results as 75% and >10% of curcumin were found in mice feces and bile, respectively [408]. In 1980, Ravindranath and Chandrasekhara showed that although 400 mg of curcumin was orally administered to rats, the presence of curcumin was not detected in heart blood while only traces amount of curcumin was present in portal blood (< 5 μg/mL), liver and kidney (< 20 μg/tissue) after 15 min to 24 h of administration [406]. Using radiolabeled curcumin, the same group further examined the metabolism route of curcumin with doses ranging from 10 to 400 mg. The radioactivity was the highest in the blood and followed by liver and kidney [409]. The major elimination route was found to be through feces and almost none was found in urine. At low doses (10 and 80 mg) of [^3^H]-curcumin, almost all labeled-curcumin was excreted within 72 h while considerable amount of the high dosage (400 mg) of labeled curcumin were retained in tissue for 12 days [409]. The pharmacokinetics of curcumin in mice was compared using either oral or intraperitoneal administration [410]. With the administration of curcumin orally at 0.1 g/kg, the level of curcumin in plasma reached the highest concentration (0.22 μg/mL) at 1 h; then dramatically decreased to below 5 ng/mL within 6 h. Surprisingly, when curcumin administered through intraperitoneal (0.1 g/kg), the curcumin in plasma concentration reached maximal at 2.25 μg/mL but decreased rapidly within 1 h. Meanwhile, the distribution of curcumin was mainly concentrated in intestine (>100 μg/g), followed by spleen (26.1μg/g), liver (26.9 μg/g), kidney (7.5 μg/g) and brain tissue (0.4 μg/g) [410]. Later, Perkins *et al*. evaluated the pharmacokinetics of curcumin administered to Min/+ mouse model of FAP either through dietary or *via *intraperitoneal route as a single dose of radiolabeled curcumin. The small intestinal mucosa contained different levels of curcumin ranging from 39 to 240 nmol/g, but curcumin level in plasma was almost below the detection limit (5 pmol/mL) [411]. A recent report by Yang *et al.* showed that the maximal curcumin level in serum was 0.36 ± 0.05 µg/mL when 10 mg/kg of curcumin was given to rats through intravenous administration compared to a mere 0.06 ± 0.01 µg/mL curcumin in serum level when administered orally although the dose was 50 folds higher in oral administration [412]. Based on the results of these studies, it is clear that administration route plays important in serum level of curcumin, in which curcumin bioavailability is much lower when administered orally compared to intraperitoneal and intravenous routes. 

Clinical trial studies demonstrated that low systemic bioavailability of curcumin is observed in human following oral administration; attributable to the high metabolic rates of liver and intestine in which the dosages of curcumin ranging between 5-50 μM were rapidly cleared from gastrointestinal systems within few hours after administration. The first phase I clinical trial to study the toxicology, pharmacokinetics and biological effects of curcumin on human were conducted with 25 patients with high risk or pre-malignant lesions [407]. Patients were initially administered with the starting dose of 500 mg/day of curcumin and if no apparent toxicity occurred, increments in dosage were done step-wise in the order of 1000, 2000, 4000, 8000 to 12000 mg/day. However, the planned increment to 12000 mg/day was not carried out as the bulk volume of curcumin was not well tolerated in the patients beyond 8000 mg/day dose. The serum concentrations of curcumin were the highest for the first 2 h and gradually decreased within 12 h. The average curcumin concentrations after oral intake of 4000 mg, 6000 mg and 8000 mg were 0.51 ± 0.11 μM, 0.63 ± 0.06 μM and 1.77 ± 1.87 μM, respectively. Their main finding showed that oral intake of up to 8000 mg/day curcumin for 3 months is well tolerated by patients [407].

Sharma *et al*. conducted a pilot study of colorectal cancer patients with *Curcuma* extract which contained 36–180 mg curcumin in proprietary capsule for 4 months. Neither curcumin nor its metabolites were detected in the plasma, blood and urine up to 29 days of oral intake treatments. However, traces of curcumin and curcumin sulfate were detected in feces. Their study showed that the administration of 36 mg curcumin for 29 days resulted in 59% decrease in glutathione *S*-transferase activity while the level of oxidative DNA adduct-3-(2-deoxy-β-di-erythro-pentafuranosyl)-pyr[1,2-α]-purin-10(3*H*) one (M_1_G) was constant throughout the experiment. Their results suggested that the tolerated dose of curcumin in patients was 2.2 g/day of *Curcuma* extract, which is equivalent to 180 mg of curcumin [413]. 

In another study, the daily oral intake of 3600 mg curcumin by colorectum cancer patients was shown to be sufficient to elicit pharmacological effect as measured by the effect on M_1_G and cyclooxygenase-2 (COX-2) expression. In their clinical report, patients with colorectal cancer ingested daily doses of 450, 1800 and 3600 mg curcumin for a week prior to surgery. The level of M_1_G decreased from 4.8 ± 2.9 to 2.0 ± 1.8 adducts per 107 nucleotides in malignant colorectal tissues for patients ingesting 3600 mg curcumin. In concurrent with other clinical reports, the curcumin and its metabolites in plasma were unquantifiable even after administrating a dose as high as 3600 mg. This further supported the hypothesis of low systemic curcumin bioavailability when administered orally. It should be noted that while ingested curcumin is cleared rapidly, which is considered disadvantageous, however the amount of administered curcumin (3600 mg daily) conferred sufficient anti-oxidant activity in human intestinal tract to result in pharmacodynamic changes corresponding to intestinal chemopreventions [414, 415].

### DNA Damage and Chromosomal Alterations

Curcumin is known to exert chemopreventive activities towards a wide spectrum of cancer cell lines, usually active in various stages such as cell proliferation, metastasis and angiogenesis [61]. Unfortunately, recent accumulating studies have in fact pointed to the contrary and damaging effects, raising concern regarding the safety of curcumin. In the light of study by Goodpasture and Arrighi, it hinted on the possibility of curcumin being harmful to certain mammalian cells by inducing chromosome deviations at concentrations as low as 10 μg/mL [420]. Several *in vitro *and *in vivo *studies have since then shed light on DNA damage and chromosomal mutations induced by curcumin at concentrations near to those that exerted pharmacological and chemopreventive effects [421-423]. It is becoming increasingly clear that curcumin exhibited both conflicting pro- and anti- oxidants properties as evident from their stimulation of quercetin-induced nuclear DNA damage, lipid peroxidation and protein damage, which was escalated in the presence of Cu or Fe [424]. It was further exemplified from the study by Ahsan and Hadi whereby in the presence of Cu^2+^, curcumin bound and caused strand scission in DNA. They showed that curcumin directly produced ROS such as H_2_O_2_, O_2_^-^ and OH^-^ which were responsible in DNA cleavage activities [423]. Kelly *et al*. reported similar findings which highlighted DNA damage in Jurkat T-lymphocytes by curcumin which were linked to series of mechanisms involving the generation of ROS [425]. In another study, DNA fragmentation and base damage was induced by curcumin in concurrent presence of Cu and isozymes of cytochrome p450 (CYP) present in lung, lymph, liver and skin [421]. The damage was possibly due to *O*-demethylation of curcumin to form *O*-demethyl curcumin radicals as catalyzed by CYP which in turns, with Cu, forms DNA-damaging Cu^+^-hydroperoxo complex [421]. Meanwhile, Yoshino *et al*. reported the formation of copper-dependent 8-hydroxy-deoxyguanosine in response to curcumin, which is believed to be the cause of DNA damage and apoptotic cell death [426]. Evidence from a study conducted by Cao *et al*. showed the damages of both DNA and mitochondrial genomes in human hepatoma G2 cells induced by curcumin were dose-dependent in which the damage was more pronounced in mitochondria [266]. The hypothesis regarding the dual conflicting roles of curcumin in carcinogenesis is further strengthened from their study as the lack of DNA damage was observed at low curcumin dose while elevated oxidative stress, DNA damage and the onset of cell death was evident at higher concentration of curcumin. It was suggested that the elevation of ROS and lipid peroxidation levels generated directly or indirectly by curcumin is possibly responsible for the deteriorating states of DNA in human hepatoma G2 cells [266]. Meanwhile, Strasser *et al*. confirmed that curcumin increased the ROS level in U937 cells [427]. Recently, Li *et al*. demonstrated that relatively low concentrations of curcumin elicited significant increase in induction of DNA damage in lung epithelial cells [428]. 

These negative and damaging effects of curcumin demonstrated by numbers of studies seemed to share a common trait: elevated levels of ROS that serves as promoter to carcinogenesis. This suggestion was made based on the following facts: (a) cancer cells had elevated level of ROS compared to normal cells, (b) malignant phenotype of cancer cells can be reversed by lowering the cellular level of ROS and (c) the presence of ROS induces cell malignant transformation. This effect is visible only when high concentration of curcumin was used while at lower concentration, curcumin still behaves as a potential anti-oxidant agent. It is known that α,β-unsaturated ketone in curcumin reacts covalently with the thiol groups of cysteine residues of proteins through a reaction Michael addition. This could answer the reason why curcumin generates ROS by irreversibly modifying the anti-oxidant enzyme thioredoxin reductase, inducing topoisomerase II-mediated DNA damage and inactivating the tumor suppressor protein. 

### Curcumin Metabolites

**Fig. (11):**
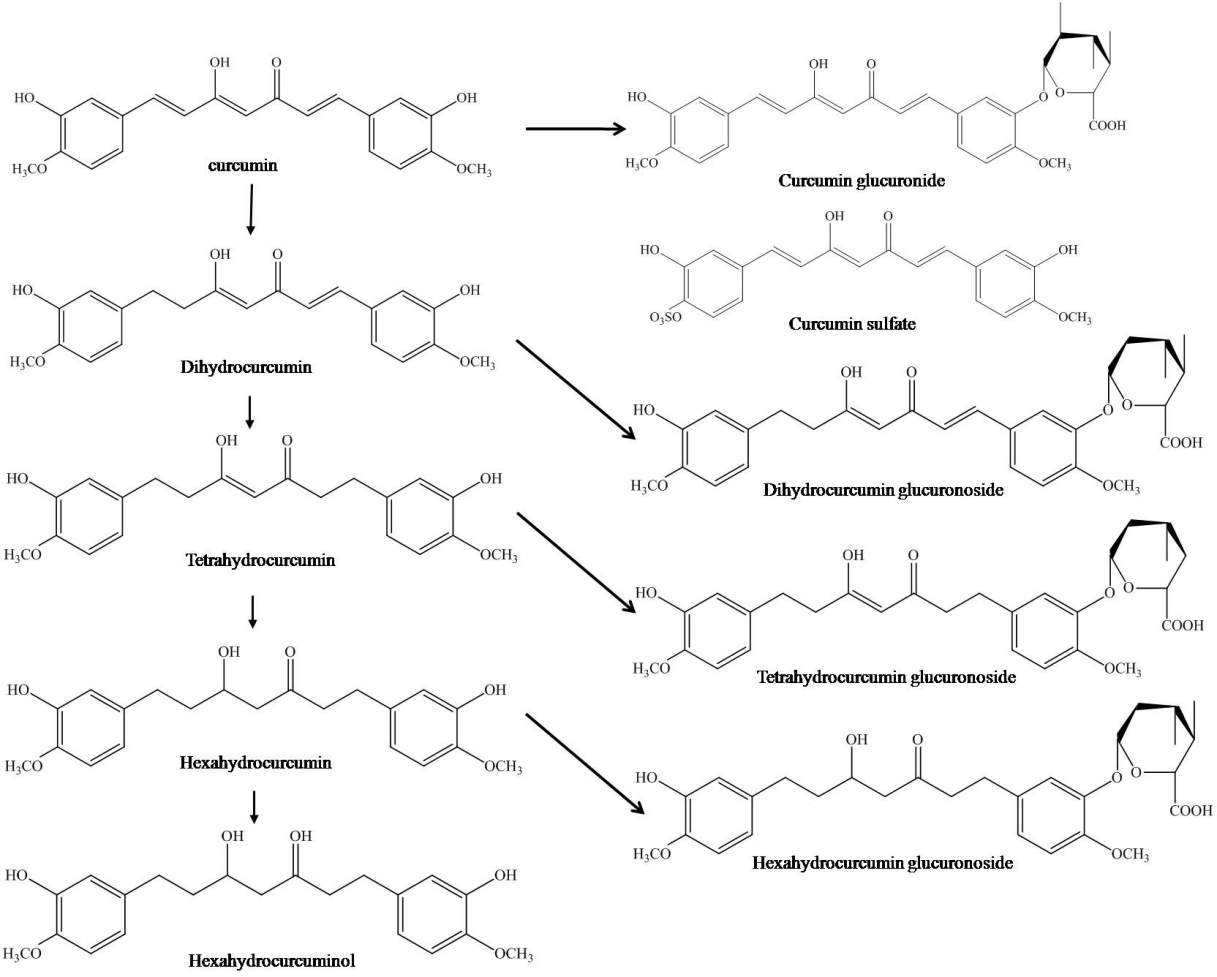
Major metabolites of curcumin.

Numbers of reports demonstrated that curcumin could be subjected to conjugations such as sulfation and glucuronidation in different types of tissues. In 1980, Ravindranath and Chandrasekhara, sulphate conjugates and glucuronide were found in the mice’s urine after oral administration of 400 mg curcumin. Glucuronide concentration was maintained at high level for more than six days and unirary sulfate level was higher than normal level for more than 40 days [406]. According to Holder *et al.,* glucuronides, tetrahydrocurcumin and hexahydroxycurcumin were the major billiary metabolites of curcumin in mice [408]. Later, Pan *et al*. proposed the possibility of biotransformation and metabolites of curcumin based on mice model. Glucuronide conjugates such as curcumin-glucuronoside, dihydrocurcumin-glucuronoside and tetrahydrocurcumin-glucuronoside as well as tetrahydrocurcumin were the major metabolites of curcumin in mice model [410] (Fig. **11**). These results were in agreement with those of Ireson *et al*., who studied the metabolites of curcumin in human and rats [429, 430]. In addition to tetrahydrocurcumin and hexahydroxycurcumin, Hoehle *et al.* found that octahydrocurcumin is another formation of reductive metabolites of curcumin in rat liver tissue whereby male rats had higher level of ctahydro-curcumin compared to female rats [431]. 

## FUTURE OUTLOOK OF CURCUMIN IN NEURO-DEGENERATIVE DISEASES

The accolade and credentials of curcumin as a multi-functional therapeutic compound in neurodegenerative diseases, cardiovascular, malignancies and other diseases have been supported by extensive published studies over the few decades. The therapeutic effects of curcumin were further strengthened in epidemiological evidences in curcumin-consuming populations such as India whereby long term consumption of curcumim showed remarkably lower incidence rate of neurodegenerative cases. The outcomes of numerous laboratory data performed in *in vivo *and *in vivo* studies have made it clear that curcumin also posed minimal toxicity towards neuronal cells. Although experimental laboratory results seem very promising, data on human clinical trial is limited. Data collected from numerous studies over the last thirty years stated that a multi-targeted and pleotropic therapy showed higher success in curing neurodegenerative diseases as opposed to a mono-targeted therapy. For instance, in the case of treating AD and PD, therapeutic strategies earmarked include compounds with anti-inflammatory, anti-amyloid deposition and anti-oxidant properties. In addition, these compounds should be tolerable (non-toxic) even in high doses, orally available, low cost and easily accessible. As a result, compounds with multiple cellular targets such as curcumin become the subject of intense research works. As mentioned in the review, curcumin could modulate multiple signaling pathways involved in virtually most neurodegenerative diseases and effectively treat neurodegenerative diseases as an improved therapeutic modality. Secondly, curcumin could easily cross the tight junction of BBB as this is important to ensure effective pharmacokinetic actions in CNS. Thirdly, low bioavailability and pre-mature degradation of curcumin resulting in sub-therapeutic levels remains the main disadvanteges of curcumin therapy that poses great challenges to date. As discussed in the review, several studies have showed that curcumin is insoluble in aqueous solution, extremely unstable in alkaline condition, easily degraded and metabolized by human body. Recent trend showed a large surge of scientific data reporting the conversion of water-insoluble curcumin into nano-sized particles, which greatly improved curcumin’s solubility and consequently its bioavailability *in vivo *[432, 433]. Significant improvement in cell uptake and longer circulation time of curcumin in human is achieved *via *coating with special biopolymers [434]. As majority of therapeutic data of curcumin are based on *in vitro *and *in vivo *model, more extensive and well-controlled human trials should be established to accurately determine its optimal administration dose, route of administration and interactions with different organs.

## Figures and Tables

**Table 1.** Curcumin in Traditional Medicine and Modern Scientific Applications

Curcumin [1,7-bis-(4-hydroxy-3-methoxy-phenyl)-hepta-1,6-diene-3,5-dione]	
Traditional application	Modern application
Rheumatism	Alzheimer
Skin diseases	Parkinson's disease
Body aches	Multiple sclerosis
Wounds dressing	Epilepsy
Intestinal worms	Cerebral injury
Diarrhoea	Mangolian Gerbils
Intermittent fevers	Cardiovascular diseases
Hepatic disorders	Allergy
Biliousness	Asthma
Urinary discharges	Bronchitis
Dyspepsia	Rheumatism
Inflammation (including colic inflammation)	Inflammation (including bowel disease and colitis)
Constipation	Renal ischemia
Leukoderma	Diabetes
Amenorrhoea	AIDS
Eye infections	Gastrointestinal cancers
Burns	Liver cancers
Acne	Lung cancers
Sprains	Blood cancers
Swelling	Breast cancers
Asthma	Oral cancers
Allergy	Prostate cancers
Hyperactivity	Skin cancers
Diabetic	Colon cancers
Cough	Ovary cancers
Sinusitis	Pancreas cancers
Flu	Head and neck cancers
Liver disorder	Brain cancers
Abdominal pain	Gastric cancers

**Table 2.** The Properties of Curcuminoids

Chemical Name	Common Name	Physicochemical Properties			
		Molecular Mass (g/mol)	Melting Point (°C)	Solubility in Hexane or Ether	Solubility in Alcohol or Acetone
di-cinnamoyl methane	Curcumin	368.4	183.0-186.0	Insoluble	Soluble
4-hydroxy cinnamoyl (feruloyl) methane	Demethoxycurcumin	338.0	172.5-174.5	Insoluble	Soluble
*bis*-4-hydroxy cinnamoyl methnane	Bisdemethoxycurcumin	308.1	224.0	Insoluble	Soluble

**Table 3.** Toxicity Effect of Curcumin Against Bacteria, Parasites and Viruses

Bacteria	Refs
Gram positive	
*Bacillus subtilis*	[84]
*Bifidobacterium. Longum *BB536	[85]
*Bifidobacterium*. *Pseudocatenultum *G4	[85]
*Eenterococcus faecalis*	[85, 86]
*Lactobacillus casei *shirota	[85]
*L. acidophilus*	[85]
*Sarcina lutea*	[86]
*Staphylococcus intermedius*	[86]
Gram negative	
*Escherichia coli*	[85]
*Helicobacter pylori*	[91, 92]
*Neiserria gonorrhoeae*	[93]
*Salmonella *sp.	[94]
*Staphylococcus aureus*	[86, 95]
*Mycobacterium tuberculosis*	[96]
Parasites	
*Leishmania donovani*	[97-100]
*L. major*	[98, 99]
*L. tropica*	[99]
*L. infantum*	[99]
*Giardia lamblia*	[101]
*Plasmodium falciparum*	[102]
*Trypanosoma brucei*	[101, 103]
Virus	
Hepatitis C	[104]
Hepatitis B	[105]
Human T-cell leukemia type-1	[106]
Human papiloma	[107]
Coxsackie B3	[108]
Japanese encephalitis	[109]
Human immunodeficiency type-1	[110]

**Table 4.** Biodistribution of Curcumin in Human, Rat and Mice Model with Different Doses and Administration Routes

Location of Curcumin	Dose	Detection Level	Model	Route	Refs
Plasma	100 mg/kg	2.25 µg/mL	Mice	Intraperitoneal	[410]
	100 mg/kg	0.22 µg/mL	Mice	Oral	[410]
	100 mg/kg	25 ± 2 nmol/mL	Mice	Intraperitoneal	[410]
	500 mg/kg	0.06 ± 0.01 µg/mL	Rat	Oral	[412]
	10 mg/kg	0.36 ± 0.05 µg/mL		Intravenous	[412]
	3.6 g/day	11.1 ± 0.6 nmol/mL	Human	Oral	[413]
Serum	340 mg/kg	6.5 ± 4.5 nM	Rat	Oral	[416]
	1 g/kg	0.5 µg/mL	Rat	Oral	[417]
	2 g/kg	1.35 ± 0.23 µg/mL	Rat	Oral	[418]
	2 g/kg	0.006 ± 0.005 µg/mL	Human	Oral	[418]
	4-8 g	0.4-3.6 µM	Human	Oral	[407]
	10 g	50.5 ng/mL	Human	Oral	[419]
	12 g	51.2 ng/mL	Human	Oral	[419]
Organs					
Brain	100 mg/kg	0.4 µg/g	Mice	Intraperitoneal	[410]
	100 mg/kg	2.9 ± 0.4 nmol/g	Mice	Intraperitoneal	[411]
Liver	100 mg/kg	26.9 ± 2.6 µg/g	Mice	Intraperitoneal	[410]
	100 mg/kg	73 ± 20 nmol/g	Mice	Intraperitoneal	[411]
Kidney	100 mg/kg	7.5 ± 0.08 µg/g	Mice	Intraperitoneal	[410]
	100 mg/kg	78 ± 3 nmol/g	Mice	Intraperitoneal	[411]
Spleen	100 mg/kg	26.1 ± 1.1 µg/g	Mice	Intraperitoneal	[410]
Lungs	100 mg/kg	16 ± 3 nmol/g	Mice	Intraperitoneal	[411]
Muscle	100 mg/kg	8.4 ± 6 nmol/g	Mice	Intraperitoneal	[411]
Stomach	2 g/kg	53.3 ± 5.1 µg/g	Rat	Oral	[406]
Intestine	100 mg/kg	117 ± 6.9 µg/g	Mice	Intraperitoneal	[410]
Small intestine	2 g/kg	58.6 ± 11.0 µg/g	Rat	Oral	[406]
Large intestine	2 g/kg	5.1 ± 2.5 µg/g	Rat	Oral	[406]
Intestinal mucosa	100 mg/kg	200 ± 23 nmol/g	Mice	Intraperitoneal	[411]
Cecum	2 g/kg	51.5 ± 13.5 µg/g	Rat	Oral	[406]
Colorectum	0.4-3.6 g/kg	7-20 nmol/g	Human	Oral	[415]
